# A systematic review on the effects of resistance and plyometric training on physical fitness in youth- What do comparative studies tell us?

**DOI:** 10.1371/journal.pone.0205525

**Published:** 2018-10-10

**Authors:** Matti Peitz, Michael Behringer, Urs Granacher

**Affiliations:** 1 German Research Center of Elite Sport—momentum, German Sport University Cologne, Cologne, Germany; 2 Institute of Sports Sciences, Goethe University Frankfurt, Frankfurt, Germany; 3 University of Potsdam, Faculty of Human Sciences, Division of Training and Movement Sciences, Potsdam, Germany; National Center of Medicine and Science in Sport, TUNISIA

## Abstract

**Introduction:**

To date, several meta-analyses clearly demonstrated that resistance and plyometric training are effective to improve physical fitness in children and adolescents. However, a methodological limitation of meta-analyses is that they synthesize results from different studies and hence ignore important differences across studies (i.e., mixing apples and oranges). Therefore, we aimed at examining comparative intervention studies that assessed the effects of age, sex, maturation, and resistance or plyometric training descriptors (e.g., training intensity, volume etc.) on measures of physical fitness while holding other variables constant.

**Methods:**

To identify relevant studies, we systematically searched multiple electronic databases (e.g., PubMed) from inception to March 2018. We included resistance and plyometric training studies in healthy young athletes and non-athletes aged 6 to 18 years that investigated the effects of moderator variables (e.g., age, maturity, sex, etc.) on components of physical fitness (i.e., muscle strength and power).

**Results:**

Our systematic literature search revealed a total of 75 eligible resistance and plyometric training studies, including 5,138 participants. Mean duration of resistance and plyometric training programs amounted to 8.9 ± 3.6 weeks and 7.1±1.4 weeks, respectively. Our findings showed that maturation affects plyometric and resistance training outcomes differently, with the former eliciting greater adaptations pre-peak height velocity (PHV) and the latter around- and post-PHV. Sex has no major impact on resistance training related outcomes (e.g., maximal strength, 10 repetition maximum). In terms of plyometric training, around-PHV boys appear to respond with larger performance improvements (e.g., jump height, jump distance) compared with girls. Different types of resistance training (e.g., body weight, free weights) are effective in improving measures of muscle strength (e.g., maximum voluntary contraction) in untrained children and adolescents. Effects of plyometric training in untrained youth primarily follow the principle of training specificity. Despite the fact that only 6 out of 75 comparative studies investigated resistance or plyometric training in trained individuals, positive effects were reported in all 6 studies (e.g., maximum strength and vertical jump height, respectively).

**Conclusions:**

The present review article identified research gaps (e.g., training descriptors, modern alternative training modalities) that should be addressed in future comparative studies.

## Introduction

For many years, the effects of resistance and plyometric training in youth and its potential benefits and harms were among the most debated research topics in exercise science and physiology. During the 1970s and 80s, researchers and scientific societies postulated an increased risk of sustaining injuries when conducting resistance training because of the immaturity of the skeletal system [1–3]. In addition, it was argued that resistance training in youth is ineffective due to a lack of circulating anabolic hormones [4]. By contrast, nowadays resistance and plyometric training is deemed to be a crucial component of a health promoting lifestyle in young individuals [5]. There is compelling evidence that resistance and/ or plyometric training improves muscular fitness (i.e., muscular strength, muscular power, local muscular endurance) [6–10], bone mineral accrual [11–13], body composition [12,14], motor performance skills [15–17], and lipid profiles [1]. Furthermore, muscular fitness is positively associated with the subjective evaluation of the own worth regarding self-esteem [18]. The current position statement on youth resistance training of the UK Strength and Conditioning Association even concludes that children and adolescents may increase their risk for negative health outcomes during adulthood if they do not participate in physical activities that build up strength and improve motor performance skills [12].

Despite today’s compelling evidence for the effectiveness and safety of youth resistance and plyometric training, there is still inconclusive evidence regarding the effects of different moderator variables on physiological adaptive processes following resistance and plyometric training programs in youth. Previous meta-analyses tried to address this question by means of meta-regressions and subgroup analyses. For example, a previously published meta-analysis revealed that resistance training induced strength gains only slightly increased with chronological age [6]. The same meta-analysis showed that the intervention period and the number of performed sets positively affected the outcome. In another meta-analysis, it was found that resistance training induced improvements in fundamental and sport-specific movement skills significantly decreased with increasing chronological age of the participants [15]. By contrast, no other training modalities describing the volume (e.g., number of repetitions) or the intensity (e.g., the percentage rate of the 1 repetition maximum; 1RM) of the applied training significantly affected the respective outcomes of these meta-analyses.

The difficulty in identifying relevant moderator variables from meta-analyses may reflect the inherent flaws of this methodological approach. A common limitation of meta-analyses is that a wide variety of participants, interventions, and outcome measures are included in one analysis, often denoted as “mixing oranges and apples”. Even though random effects models adjust for some of the resulting statistical heterogeneity, important dissimilarities between studies get lost and may lead to erroneous conclusions. Additionally, research limitations (e.g., inappropriate description of the exercise stimulus, use of multiple jump types) as demonstrated by Ramirez-Campillo et al. for plyometric training research, further hamper the identification of these moderator variables (e.g., plyometric training volume) [19]. By contrast, individual studies that examine the effects of such variables by comparing different subgroups (from now on referred to as comparative studies), evade this issue by adjusting the variable of interest. We, therefore, reviewed comparative intervention studies that assessed the effects of different age groups, maturity levels, resistance and plyometric training descriptors, and sex on measures of physical fitness while holding other variables constant. Thus, the present review aimed at exploring the impact of different independent variables on the effectiveness of resistance and plyometric training to improve measures of physical fitness in untrained and trained children and adolescents by systematically analysing results from comparative studies. We expected this approach to shed further light on the influence of moderator variables (e.g., sex) on resistance and plyometric training related outcomes and that the results could differ from those of previously published meta-analyses [6,10,15].

## Methods

### Definitions

In the present systematic literature review, the term ‘children’ refers to boys and girls who are pre-PHV, whereas ‘adolescents’ refers to around- and post-PHV boys and girls. The term ‘youth’ includes both children and adolescents. The terms ‘trained’ and ‘untrained’ refer to the training history of each individual or the respective exercise group. In this context and in accordance with Rhea et al. [20], ‘untrained’ refers to children or adolescents who have not consistently been exposed to structured plyometric or resistance training for a minimum of one year. The term ‘trained’ refers to youth who have been performing structured plyometric or resistance training for a minimum of one year. Of note, if individuals participated in regular soccer training or any type of sport-specific training without having had any experience or history in structured resistance training, they were classified as ‘untrained’. ‘Training type’ refers to a specific form of training (e.g., resistance training or plyometric training), whereas “training descriptor” specifies the training stimulus of a certain training type (e.g., volume, intensity). The term ‘resistance training’ refers to all training methods that require the muscles to contract against an opposing force. The opposite force may be generated by free weights (e.g., barbells and dumbbells), resistance training machines, or the own body weight. This includes traditional strength training as well as ‘power training’. The latter refers to all training methods that aim at increasing the muscle's ability to contract as fast and as forcefully as possible against an external resistance. For practical reasons, this includes resistance training methods that focus on generating high movement velocities (e.g., Olympic Weightlifting) without taking advantage of the stretch-shortening cycle. Plyometric training largely differs from conventional resistance training regarding movement velocity during the execution of exercises. Eccentric and concentric muscle actions are performed in direct sequence during braking and push-off phases of plyometric jump exercises to take advantage of the stretch-shortening cycle. Therefore, the training stimulus largely differs between both training types, which is why the effects of maturation or sex on plyometric training related outcomes (e.g., jump performance) may differ from effects following conventional resistance training. The combination of resistance and plyometric training will further be described as complex training.

### Literature search

To identify relevant studies, two investigators (MP, MB) independently performed a systematic literature search between September 2017 and March 2018 using the electronic databases PubMed (1966), Web of Science (1945), and Google Scholar from their inception until March 2018. We applied a Boolean search strategy using the operators AND, OR, NOT in combination with the following keywords and truncation technique using asterisks: child*, adolescent*, youth, boy*, girl*, maturity, athlete*, strength, resistance, weight, plyometric*, weight-bearing, training, and exercise. Additionally, reference lists of identified studies, reviews, and meta-analyses were examined to identify further relevant studies. Any discrepancies regarding eligibility of studies to be included in this systematic review were resolved by a third investigator (UG). Only studies were included for further analyses if participants were healthy trained or untrained children and/or adolescents (mean age under 18 years). Of note, studies were eligible for inclusion if the study design included a comparison of different training regimes, or the same training was applied to different cohorts (age group, sex, maturational status, training status/years). Further, the included studies had to examine a physical fitness component before and after the training intervention. Additionally, comparative studies that examined the effects of complex training were excluded from this review because with complex training it is not possible to ascribe the observed training effects to either resistance or plyometric exercises. In addition, our search retrieved a small number of complex training studies which did not allow independent sub-analysis according to moderator variables.

Relevant information from the included studies, such as participant characteristics, training protocols, and study outcomes were extracted by two independent reviewers (MP, MB). We decided not to apply a risk of BIAS scale in this systematic review due to various reasons. First, Cochrane Collaboration recently recommended not to use these scales [21]. Second, the blinding of study participants and observers is difficult to achieve in training intervention studies [22]. Third, it has previously been reported that most youth resistance and plyometric training studies are of low to medium quality [17,23,24]. This would prevent the analysis of systematic reviews and meta-analyses in the field. Data are presented as means and standard deviations (Tables 1–13).

**Table 1:** Influence of the moderating variable ‘maturation’ on resistance training induced improvements in components of physical fitness.

Author	Number of subjects, sex	Age [years]	Training experience, sport-specific background	Maturity Assessment	Comparator	Frequency [sessions/week]	Duration [week]	Training Type	Training effects
Gabbett et al. 2008 [34]	35 M	14.1±0.2 16.9±0.3	UT, Rugby	n/a	G1: around-PHV G2: post-PHV	3	10	RT, COND, rugby specific	G1>G2: chin-up mENDUR (G1:↑71% G2: 219450% p < .05) G2>G1: multi-effort VJ (G1: ↑8,5% G2: ↑9,9% p < .05) G1 = G2 (p>.05): push-up mENDUR (G1:↑25% G2: ↑34%), sit-up mENDUR (G1:↑18% G2: ↑16%)
Meylan et al. 2014 [29]	33 M	12.4±0.7 13.6±0.6 14.3±0.7	n/a, Sport Academy	PHV	G1: pre-PHV G2: around-PHV G3: post-PHV	2	8, 8 detraining	RT	time x group interaction n/a G2>G1 (MBI): #↔power (G1:11% G2:16%), Fmax (#↓G1:-2.1% #?G2:2.5%), #↑10m sprint (G1:2.6% G2:4.7%), #↑30m sprint (G1:2.1% G2:3.6%) G3>G1 (MBI): 1RM (#↔G1:3.6% #↑G3:10%), power (#↑G3:20%), Fmax (#↔G3:8.7%), 10m sprint (#↑G3:4%), 30m sprint (#↑G3:3%) G3>G2 (MBI): 1RM (#↔G2:3.5%), power G2>G3 (MBI): 30m sprint G1?G2?G3 (MBI): horizontal jump (#↑G1:6.5% #↑G2:6.8% #↔G3:7.4%), Vmax (#↔G1:16% #↑G2:14% #↑G3:11%) detraining: G2&G3>G1 (MBI): 1RM (#↓G1:-4.6% #↔G2:0% #↓G3:-0.7%), #↓power (G1: -11% G2: -3% G3: -6%) G2>G1&G3 (MBI): 10m sprint (#↔G1:-0.1% #↔G2:0.4% #↓G3:-0.6%) G2>G3 (MBI): 30m sprint (#↔G2:0.4% #↓G3:-0.6%) G1?G2?G3?: Vmax (#?G1:-2% #?G2:4% #↓G3:-10%), horizonal jump (#?G1:2.4% #?G2:1.9% #↑G3:3.7%)
Steinmann 1990 [35]	192 M	11.3± n/a 14.3±n/a	n/a	n/a	G1a: pre-PHV 1x/W G1b: pre-PHV 2x/W G2a: around-PHV 1x/W G2b: around-PHV 2x/W	1/2	8	RT	"G2a>G1a &G2b>G1b" (p = n/a): 1RM bench press (G1a: ↑12% G2a: ↑10%, G1b: ↑20% G2b: ↑20%), 1RM squat (G1a: ↑17% G2a:↑13%, G1b: ↑33% G2b: ↑27%), VJ (G1a: ↑5% G2a: ↑6%, G1b: ↑7% G2b: ↑8%) "G1a = G2a & G1b = G2b" (p = n/a): 20m sprint* (G1a: ↑2% G2a: ↑1%, G1b: ↑5% G2b: ↑4%), multi-hop (G1a: ↑3% G2a: ↑2%, G1b: ↑7% G2b: ↑4%), MB pass (G1a: ↑8% G2a: ↑5% G1b:↑16% G2b: ↑11%), MB throw (G1a:↑6% G2a: ↑5% G1b: ↑17% G2b:↑10%)
Vrijens 1978 [4]	28 M	10.4±n/a 16.6±n/a	n/a	Tanner	G1: pre-pubescent G2: post-pubescent	3	8	RT	time group interaction n/a "G2>G1": MVC arm flexion (↔G1: p>.05 ↑G2: p < .02), MVC arm extension (↔G1: p>.05 ↑G2: p < .05), MVC leg flexion (↔G1: p>.05 ↑G2: p < .01), MVC leg extension (↔G1: p>.05 ↑G2: p < .02) "G1 = G2": MVC trunk flexion (↑G1: p < .01 ↑G2: p < .02), MVC trunk extension (↑G1: p < .01 ↑G2: p < .02)
Lillegard et al. 1997 [33]	91 M&F	M 11.2±1.1 F 9.5±1.4 M 14±1.0 F 13.8±3.0	n/a	Tanner	G1: Tanner 1–2 G2: Tanner 3–5	3	12	RT	G1 = G2 (p>.05): ↑10 RM triceps extension, ↑10 RM bench press, ↑10 RM lat pull, ↑10 RM leg extension
Pfeiffer & Francis 1986 [36]	64 M	10.3±1.2 13.1±1 19.8±1.2	n/a	Tanner	G1: pre-pubescent (Tanner1) G2:pubescent (Tanner 2–4) G3: post-pubescent (Tanner 5)	3	9	RT	% change in torque/kg body weight: G1>G2: knee flexion right limb at 30°/s (p < .05), G1>G2&G3: elbow flexion left limb at 120°/s (p < .05), knee extension left limb at 120°/s (p < .02), G1 = G2 = G3 (p>.05): 13/16 tests, ↑arm flexion & ↑extension at 30°/s & 120°/s, ↑ knee extension at 30°/s & 120°/s, ↔ knee flexion at 30°/s & 120°/s
Radnor et al. 2016 [37]	80 M	PLYO 12.7±0.3 RT 12.6± 0.3 CT 12.7±0.3 PLYO 16.4±0.2 RT 16.3±0.3 CT 16.2±0.3	UT	PHV	G1a: pre-PHV PLYO G1b: pre-PHV RT G1c: pre-PHV CT G2a: post-PHV PLYO G2b: post-PHV RT G2c: post-PHV CT	2	6	RT, PLYO, CT	time x group interaction for maturity n/a G1c>G1b (p < .05): 10m sprint (G1b:↔*1,1% G1c:↑*3,3%), max velocity (G1b:↔*0,4% G1c:↑*2,7%) G1a>G1b (p < .05): max. velocity (G1a:↑*2,8% G1b:↔*0,4%) G2a>G2b (p < .05): RSI (G2a:↑*4,6% G2b:↔*0,7%) G2b>G2a (p < .05): 10m sprint (G2a:↔*0,4% G2b:↑*1,8%), SJ (G2a:↔*1,4% G2b:↑*7,7%) G2c>G2a: 10m sprint (G2a:↔*0,4% G2c:↑*2,7%), SJ (G2a:↔*1,4% G2c:↑*12,9%) G2c>G2b: max velocity (G2b:↔*0,6% G2c:↑*3,9%), SJ (G2b:↑*7,7% G2c:↑*12,9%), RSI (G2b:↔*0,7% G2c:↑*3,8%)
Lloyd et al. 2015 [30]	80 M	PLYO 12.7±0.3 RT 12.6±0.3 CT 12.7±0.3 PLYO 16.4±0.2 RT 16.3±0.3 CT 16.2±0.3	UT	PHV	G1a: pre-PHV PLYO G1b: pre-PHV RT G1c: pre-PHV CT G2a: post-PHV PLYO G2b: post-PHV RT G2c: post-PHV CT	2	6	RT, PLYO, CT	G1a = G2a, G1b = G2b, G1c = G2c (p>.05): 10m sprint (↑G1a: ES = 0.38 ↑G1b: ES = 0.11 ↑G1c: ES = 0.32 ↔G2a:ES = 0.06 ↑G2b:ES = 0.36 ↑G2c:ES = 0.62), 20m sprint (↑G1a: ES = 0.45 ↔G1b: ES = 0.04 ↑G1c: ES = 0.31 ↑G2a:ES = 0.34 ↔G2b:ES = 0.08 ↑G2c:ES = 0.50), SJ (↑G1a: ES = 0.77 ↑G1b: ES = 0.52 ↑G1c: ES = 0.96 ↔G2a:ES = 0.07 ↑G2b:ES = 0.45 ↑G2c:ES = 0.79), RSI (↑G1a: ES = 0.53 ↑G1b: ES = 0.16 ↑G1c: ES = 0.19 ↑G2a:ES = 0.27 ↔G2b:ES = 0.05 ↑G2c:ES = 0.28) G1a>G2a (MBI): 10m sprint, SJ G1a: ↑4/4 tests, G1b: ↑3/4 tests, G1c: ↑4/4 tests, G2a: ↑2/4 tests, G2b: ↑2/4 tests, G2c: ↑4/4 tests
Moran et al. 2017 [38]	22 M	11.9 ± 1.2 15.0 ± 1.1	mostly UT, Swimming	PHV	G1: pre-PHV G2: post-PHV	2	8	RT	time x group interaction for maturity n/a G2>G1(MBI): ↑isometric strength mid thigh pull (G1:ES = 0.8 G2: ES = 1.3; vs. CG: G1:ES = 0.4 G2:ES = 1.7) G1>G2(MBI): VJ (↔G1:ES = 0.2 ↑G2:ES = 0.4; vs.CG: G1:ES = 1.2 G2:ES = 0.6) G1 = G2(MBI): ↔handgrip strength (G1: ES = 0.2 G2: ES = -0.3)

Note: M, male; F, female; UT, untrained; T, trained; n/a, not available; G, group; PHV, peak height velocity; PLYO, plyometric training; RT, resistance training; CT, complex training; COND, conditioning training; mENDUR, muscular endurance; RM, repetition maximum; Fmax, maximal force; Vmax, maximal velocity; VJ, vertical jump; SJ, squat jump; MB, medicine ball; MVC, maximum voluntary contraction; RSI, reactive strength index

“”, descriptive

↑, significant within-group improvement from pre to post

↔, non-significant within-group change from pre to post

MBI, interpretation based on outcomes of magnitude-based inferences

ES, effect size

#↑, substantial within-group improvements from pre to post (with >75% chance of being beneficial)

#?, unclear within-group change from pre to post

#↔, trivial change or non-substantial improvements (<75% chance of being beneficial) within-group from pre to post

#↓, impairment (>25% chance of being harmful) within-group from pre to post

**Table 2:** Influence of the moderating variable ‘sex’ on resistance training induced improvements in components of physical fitness.

Author	Number of subjects, sex	Age [years]	Training experience, sport-specific background	Comparator	Frequency [sessions/week]	Duration [week]	Training Type	Training effects
Siegel et al. 1989[39]	56M 40F	8.4±0.5	n/a	G1: M G2: F	3	12	RT	G1 = G2 (p>.05): ↑right handgrip, ↑chin- up, ↑flexed arm hang, ↑sit and reach G1>G2 (p < .02): elbow extension (G1: -1%, p>.05, G2: -8%, p < .02)
Meinhardt et al. 2013 [40]	60 M 42 F	M 12.4±1.1; F 12.0±1.1	n/a	G1: M G2: F	2	19	RT	groupxtime interaction n/a "G1 = G2": ↑smith press (G1: 38% G2: 33%), ↑1RM leg press (G1: 36% G2: 29%)
Benson et al. 2008 [41]	46M 32F	12.3±1.3	UT	G1: M G2: F	2	8	RT	G1 = G2 (p = .006): ↑bench press, ↑leg press
Vom Heede et al. 2007 [42]	29 M 31 F	10.6±n/a	n/a	G1a: M, RT G1b: M, mostly PLYO G2a: F, RT G2b: F, mostly PLYO	2	6	RT, PLYO	statistics n/a "G1a = G2a & G1b = G2b": ↑situp, ↑pushup, ↑pullup-hold, ↑LJ, ↑VJ, ↑MB throw (1 exception, but n/a)
Lillegard et al. 1997 [33]	91 M&F	M 11.2±1.1; F 9.5±1.4; M 14±1.0; F 13.8±3.0	n/a	G1: M, Tanner 1–2, Tanner 3–5 G2: F, Tanner 1–2, Tanner 3–5	3	12	RT	G1>G2: ↑10 RM lat pull (p = .02), ↑ 10RM leg extension (p = .01) G1 = G2 (p>.05): ↑ 10RM triceps extension, ↑10RM bench press, 10RM barbell curl, 10RM leg curl
Hassan 1991 [43]	18 M 20 F	M 10±0.3; F 9.8±0.3	UT	G1: M G2: F	3	6	RT	timexgroup interactions n/a "G1 = G2": ↑static & ↑dynamic rel. max force knee extension
Muehlbauer et al. 2012 [44]	13 M 15 F	M 16.8±0.8; F 16.6±0.5	UT	G1: M G2: F	2	8	RT	G2>G1: MVC (↔G1: ES = 0.9 ↑G2: ES = 1.9 p = .01), RFD(↔G1: ES = 0.3 ↑G2: ES = 2.2, p = .001) G1 = G2 (p>.05): CMJ (↑G1: ES = 0.6 ↑G2: ES = 1.4)
Letzelter & Diekmann 1984 [45]	190 M 192 F	n/a (3th & 4th grade)	UT	G1: M G2: F	2	12	RT	"G1 = G2" (p = n/a): 1RM bench press (G1: ↑12% G2: ↑10%), 1RM squat (G1: ↑10% G2: ↑10%)

Note: M, male; F, female; UT, untrained; n/a, not available; G, group; PLYO, plyometric training; RT, resistance training; RM, repetition maximum; Fmax, maximal force; VJ, vertical jump; LJ, long jump; CMJ, countermovement jump; MB, medicine ball; MVC, maximum voluntary contraction; ES, effect size

“”, descriptive

↑, significant within-group improvement from pre to post

↔, non-significant within-group change from pre to post.

**Table 3:** Influence of the moderating variables ‘training intensity and volume’ on resistance training induced improvements in components of physical fitness.

Author	Number of subjects, sex	Age [years]	Training experience, sport-specific background	Comparator	Frequency [sessions/week]	Duration [week]	Training Type	Training effects
Rarick & Larsen 1958 [46]	30 M	17±n/a	n/a	G1: 1Wdh./d @ 66% MVC G2:5-8Wdh./d @80%MVC	5	4	RT	G1 = G2 (p>.05): ↑static strength wrist flexor, static strength retention wrist flexor
Faigenbaum et al. 2001 [47]	44 M 22 F	7.8±1.4 8.5±1.6 8.3±1.6 9.2±1.6	UT	G1: 1x 6–8 (heavy) G2: 1x13-15 (moderate) G3: 1x6-8 + 6–8 MB G4: 1x 13–15 MB	2	8	RT	G2>G1 (p<05): ↑"mENDUR" chest press G3>G1&G4 (p < .05): ↑"mENDUR" chest press G2&G3>CG (p < .05): ↑1RM chest press
Faigenbaum et al. 1999 [48]	32 M 11 F	7.8±1.4 8.5±1.6	UT	G1: 1x6-8 G2: 1x13-15	2	8	RT	G1 = G2 (p>.05): mENDUR chest press, ↑1RM leg extension (G1: 31% G2: 41%) G2>G1 (p = .03): mENDUR leg extension G2>CG, G1 = CG: 1RM chest press (G1: 5%, p>.05 G2: 16%, p < .05), mEDNUR chest press
Faigenbaum et al. 2005 [49]	20 M 23F	10.4±1.2 10.4±1.5	mostly UT	G1: 1x6-10RM G2:1x 15-20RM	2	8	RT	G1 = G2(p = n/a): ↑1RM chest press (G1: 21% G2: 23%), ↑15RM leg-press (G1: 32% G2: 42%) G2>CG(p < .05): 15RM leg-press
Yuktasir & Tuncel 1998 [50]	47 M	16–17±n/a	UT	G1: 3x6@80–85% (concentric faliure) G2: 1x12@60–65% + eccentric manual resistance (concentric & eccentric faliure)	3	8	RT	G1 = G2: ↑1RM (G1: 19% G2: 19%, p>.05), ↑MVC (G1: 16% G2: 15%, p = n/a)
Steele et al. 2017 [51]	17 M 16 F	14±1	UT	G1: 2x 4–6 G2: 2x 12–15 no control group	2	9	RT	G1 = G2 (p>.05): ↑1RM bench press (G1: 15% ES = 1.64 G2: 14% ES = 1.62), ↑mENDUR bench press (G1: 46% ES = 1.66 G2: 44% ES = 1.51)
Gonzalez-Badillo et al. 2005 [52]	41 M	16.4±1.3 16.5±1.4 16.8±1.7	T	matched rel. Intensity: G1: low volume G2: medium volume G3: high Volume	4–5	10	RT	G1 = G2 = G3 (p>.05): 1RM squat (G1: ↑5%, G2: ↑4%, G3: ↑5%), 1RM Clean and Jerk (G1: ↑4%,G2: ↑4%, G3: ↑3%) G2>G1 (&G3): 1RM snatch (↑G2 ↔G1: p = .02,(↑G2 ↔G3: p = .09))
Gonzalez-Badillo et al. 2006 [53]	29 M	17.1±1.7 16.9±1.7 17.5±1.9	T	matched volume: G1: low volume of high intensity G2: medium volume of intensity G3: high volume of high intensity	4–5	10	RT	timexgroup interactions n/a "G2>G1&G3" (ES): mean total ES (G1: 0.31 G2: 0.61 G3: 0.24), 1RM Clean&Jerk (G1: ↑3% G2: ↑11% ↔G3: %n/a), 1RM Squat (G1: ↑5%, G2: ↑10%, G3: ↑7%); 1RM snatch (↔G1 ↔G2 ↔G3)

Note: M, male; F, female; UT, untrained; T, trained; n/a, not available; G, group; RT, resistance training; mENDUR, muscular endurance; RM, repetition maximum; MB, medicine ball; MVC, maximum voluntary contraction; ES, effect size

↑, significant within-group improvement from pre to post

↔, non- significant within-group change from pre to post.

**Table 4:** Influence of the moderating variable ‘training frequency’ on resistance training induced improvements in components of physical fitness.

Author	Number of subjects, sex	Age [years]	Training experience, sport-specific background	Comparator	Duration [Week]	Training Type	Training effects
Steinmann 1990 [35]	192 M	11.3± n/a; 14.3±n/a	n/a	G1a: 1x/W (11.3) G1b: 1x/W (14.3) G2a: 2x/W (11.3) G2b: 2x/W (14.3)	8	RT	G2a>G1a, G2b>G1b (p = n/a): ↑1RM bench press, ↑1RM squat, ↑ 20m sprint, ↑horizontal jump,↑ MB toss,↑MB throw G2a>G1a(p = n/a): ↑VJ G1b = G2b (p = n/a): ↑VJ
Faigenbaum et al. 2002 [54]	34 M 21 F	10.2±1.4 9.7±1.4	UT	G1: 1x/W G2: 2x/W	8	RT	timexgroup interactions between G1&G2 n/a G2>CG, G1 = CG (p = .000): 1 RM chest press (G1: ↔9% G2: ↑12% vs CG: ↔4%), G1&G2>CG (p = .009): 1RM leg press (G1: ↑14% G2: ↑25% CG: ↔2%) G1 = G2 (p>.05): ↔handgrip stength, ↔flexibility, ↔VJ, ↔LJ
Reuter 2003 [55]	195 sex n/a	11,8±n/a 15,4±n/a	n/a	G1: 1x/W G2: 2x/W	7	RT	timexgroup interactions n/a "G2>G1": maximal force: ↑bench press (G1: 21% G2: 32%), ↑latissimus pull (G1: 6% G2: 13%), ↑crunch (G1: 12% G2: 19%), ↑leg press (G1: 8% G2: 17%), mENDUR: ↑bench press (G1: 50% G2: 76%), ↑latissimus pull (G1: 68% G2: 106%), ↑crunch (G1: 50% G2: 118%), ↑leg press (G1: 73% G2: 140%)
DeRenne et al. 1996 [56]	21 M	13,3±1.3	UT, basketball	3x/W for 12 W, then 12 W: G1: 1x/W G2: 2x/W	12	RT	G1 = G2>CG (p>.05): strength retention: 1RM bench press, 1RM leg press, mENDUR pull ups
Uppal & Tunidau 1991 [57]	60 M	15±n/a	UT	G1: 2x/W G2: 3x/W G3: 5x/W	6	RT	G1 = G2 (p>.05): ↑REP pullups (G1: 31% G2: 105%), ↑REP situps (G1: 39% G2: 61%), ↑broad jump (G1: 6% G2: 7%) G2 = G3 (p>.05): ↑REP pullups (G3: 95%), ↑REP situps (G3: 64%), ↑broad jump (G3: 9%) G3>G1 (p < .05): ↑REP pullups, ↑REP situps, ↑broad jump

Note: M, male; F, female; UT, untrained; n/a, not available; G, group; CG, control group; RT, resistance training; mENDUR, muscular endurance; RM, repetition maximum; MB, medicine ball; VJ, vertical jump; LJ, long jump; REP, repetition

“”, descriptive

↑, significant within-group improvement from pre to post

↔, non- significant within-group change from pre to post.

**Table 5:** Influence of the moderating variable ‘periodization’ on resistance training induced improvements in components of physical fitness.

Author	Number of subjects, sex	Age [years]	Training experience, sport-specific background	Comparator	Frequency [sessions/week]	Duration [week]	Training Type	Training effects
Moraes et al. 2013 [58]	38 M	15.5±0.9 15.4±1.1	UT	G1: NP G2: DUP	3	12	RT	G1 = G2 (p>.05): ↑1RM bench press (G1: 19% G2: 36%), ↑1RM leg press (G1: 88% G2: 107%) "G2>G1": 1RM bench press (G1: ES = 1,2 G2: ES = 3,4), 1RM leg press (G1: ES = 5.1 G2: ES = 6.3)
Harries et al. 2016 [59]	26 M	16.8±1.0 17.0±1.1	T	G1: LP G2: DUP	2	12	RT	G1 = G2 (p>.05): ↑5RM squat (G1: ES = 1.6 G2: ES = 2.3), ↑5RM bench press (G1: ES = 0.6 G2: ES = 0.3)
Ullrich et al. 2016 [60]	5 M 6 F	14.8±0.6	T	crossover: G1: LP G2: DUP	3	2x 4	RT	G1 = G2 (p>.05): ↑1RM squat, ↑1RM bench press, ↑1RM bench pull, ↑1RMl at pull down, ↑MVC knee extension
Foschini et al. 2010 [61]	15 M 17 F	16.5±1.7	UT, obese	G1: LP G2: DUP	3	12	RT, ENDUR	G1 = G2: ↑15RM bench press (G1:175% ES = 2.78 G2:220% ES = 3.43, p = .09), ↑15RM leg press (G1: 396% ES = 6.96 G2: 455% ES = 8.25, p = .32)

Note: M, male; F, female; UT, untrained; T, trained; n/a, not available; G, group; NP, no periodization; LP, linear periodization; DUP, daily undulating periodization; RT, resistance training; ENDUR, endurance training; RM, repetition maximum; MVC, maximum voluntary contraction; ES, effect size

“”, descriptive

↑, significant within-group improvement from pre to post.

**Table 6:** Influence of the exercise mode and type on training induced improvements in components of physical fitness.

Author	Number of subjects, sex	Age [years]	Training experience, sport-specific background	Comparator	Frequency [sessions/week]	Duration [week]	Training Type	Training effects
Shields et al. 1985 [62]	53 M	16±n/a 15±n/a	UT	G1: isotonic G2: isokinetic	3	8	RT	G2>G1 (p = .001): ↑isokinetic leg press strength at 30°/s (G1: 17,4%, G2: 29,6%) G1 = G2 (p>.05): ↑MVC leg strength (G1:7,7% G2: 10,5%), ↑leg flexion mENDUR (G1: 43% G2: 43%), ↑leg extension mENDUR (G1: 31%, G2: 22%), ↑VJ (G1: 9.6%, G2:10.4%)
Smith & Melton 1981 [63]	12 M	16–18	UT	G1: isotonic variable resistance G2: isokinetic slow G3: isokinetic fast	3	6	RT	statistical analyses n/a "G1 = G2 = G3": isometric, isokinetic, isotonic leg strength "G3>G1&G2": VJ, broad jump, 40-yard sprint
Bulgakova et al. 1987 [64]	37 (sex n/a)	11–12	n/a, swimming	G1: sports specific (in water strength) G2: resistance machine	2	24	RT	G2>G1 (p = n/a): ↑dry-land strength endurance (speed strength endurance, strength endurance) G1>G2 (p = n/a): ↑max. swimming speed, swimming technique
Flanagan et al. 2002 [65]	28 M 30 F	8.8±0.5 8.6±0.5	UT	G1: machine G2: body weight	2	10	RT	G2>G1* (p < .05): MB put (G1: ↔4% G2: ↑12%) *but G1pre>G2pre G1 = G2(p>.05): ↔shuttle run (G1: 2% G2: 3%), ↔LJ (G1: 9% G2: 4%)

Note: M, male; F, female; UT, untrained; T, trained; n/a, not available; G, group; RT, resistance training; mENDUR, muscular endurance; RM, repetition maximum; VJ, vertical jump; LJ, long jump; MB, medicine ball; MVC, maximum voluntary contraction

↑, significant within-group improvement from pre to post

↔, non- significant within-group change from pre to post.

**Table 7:** Influence of the moderating variable ‘supervision’ on resistance training induced improvements in components of physical fitness.

Author	Number of subjects, sex	Age [years]	Training experience, sport-specific background	Comparator	Frequency [sessions/week]	Duration [week]	Training Type	Training effects
Coutts et al. 2004 [66]	42 M	16.6±1.2 16.8±1.0	n/a, rugby	G1: supervised G2: unsupervised	3	12	RT	G1>G2 (p < .05): ↑3RM bench press (G1:30% G2:15%), ↑3RM squat (G1:40% G2:26%), ↑max REP pull up (G1:97% G2:46%) G1 = G2 (p>.05): ↑VJ (G1:7% G2:10%), ↑10m sprint (G1:1% G2:1%), ↑20m sprint (G1:1% G2:1%)
Klusemann et al. 2012 [67]	17 M 22 F	M 14.01±1.0 F 15.0±1.0	UT, basketball	G1: supervised G2: unsupervised, online-video-based	2	6	RT	time x group interactions n/a G1>G2(MBI): Yo-Yo (G1:35% G2:10%), FMS (G1:14% G2:0.1%) G2>G1(MBI): agility (G1:2.2% G2:3.8%), anaerobic capacity/ line drill (G1:-0.5% G2:0.9%) G1 = G2 (MBI): VJ (G1:5.4% G2:4.3%), 20m sprint (G1:0.5% G2:1.6%) G1?G2: pushup-test (G1:20% G2:23%), pullup-test (G1:1% G2:1%), CMJ (G1:5% G2:-0.6%), sit and reach (G1:1.2% G2:1.6%)
Smart & Gill 2013 [68]	44 M	15.4±1.4 15.1±1.3	n/a, rugby	G1: supervised G2: unsupervised	4	15	RT, Speed, ENDUR	time x group interactions n/a G1>G2 (MBI): 1RM chin up (G1vsG2: +9.1%), 1RM bench press (G1vsG2: +16.9%), 1RM box-squat (G1vsG2:+50.4%), VJ (G1vsG2: +4.2%), 10m sprint (G1vsG1: +2.1%) G1?G2 (MBI): 20m sprint, 30m sprint, 60m sprint, 400m, 1500m

Note: M, male; F, female; UT, untrained; n/a, not available; G, group; RT, resistance training; ENDUR, endurance training; RM, repetition maximum; VJ, vertical jump; CMJ, countermovement jump; FMS, functional movement screen

↑, significant improvement from pre to post within group

MBI, interpretation based on outcomes of magnitude-based inferences

?, trivial or unclear difference between groups.

**Table 8:** Influence of the moderating variable ‘maturation’ on plyometric training induced improvements in components of physical fitness.

Author	Number of subjects, sex	Age [years]	Training experience, sport-specific background	Maturity Assessment	Comparator	Frequency [sessions/week]	Duration [week]	Training Type	Training effects
Marta et al. 2014 [69]	37 M 43 F	M 10.7±0.4 F 10.9±0.3	UT	Tanner	G1: Tanner 1 G2: Tanner 2	2	8	PLYO	G1 = G2 (p>.05): MB throw 3 kg (G1:↑9% vs G2: ↑7%), MB throw 1kg (G1: ↑7% vs G2: ↑5%), LJ (G1:↑6% vs G2: ↑5%), VJ (G1: ↑10% vs G2: ↑5%), 20m sprint (G1: ↑2% G2: ↑2%)
Moran et al. 2016 [70]	38 M	12.6±0.7 14.3±0.6	UT, hockey/ intense physical education	PHV	G1: pre-PHV G2: around-PHV	2	6	PLYO	time x group interaction n/a G1 = G2 (MBI): #↔CMJ (G1: ES = 0.0 G2: ES = 0.1), #↔30m sprint (G1: ES = -0.1 G2: ES 0.1) G2>G1(MBI): 10m sprint (#↔G1: ES = 0.1 #↑G2: ES = 0.4)
Lloyd et al. 2012 [71]	129 M	9.4±0.5 12.3±0.3 15.3±0.3	UT	n/a	G1: (early) pre-PHV G2: (late) pre-PHV G3: post-PHV	2	4	PLYO	G1 = G2 = G3 (p>.05): change absolute leg stiffness (↔G1, ↑G2, ↑G3) G2>CG (p < .05): ↑RSI, ↑absolute & ↑relative leg stiffness, ↑contact time G3>CG(p < .05): ↑ absolute & ↑relative leg stiffness, ↑ contact time
Lloyd et al. 2015 [30]	80 M	PLYO 12.7±0.3 RT 12.6±0.3 CT 12.7±0.3 PLYO 16.4±0.2 RT 16.3±0.3 CT 16.2±0.3	UT	PHV	G1a: pre-PHV PLYO G1b: pre-PHV RT G1c: pre-PHV CT G2a: post-PHV PLYO G2b: post-PHV RT G2c: post-PHV CT	2	6	RT, PLYO, CT	G1a = G2a, G1b = G2b, G1c = G2c (p>.05): 10m sprint (↑G1a: ES = 0.38 ↑G1b: ES = 0.11 ↑G1c: ES = 0.32 ↔G2a:ES = 0.06 ↑G2b:ES = 0.36 ↑G2c:ES = 0.62), 20m sprint (↑G1a: ES = 0.45 ↔G1b: ES = 0.04 ↑G1c: ES = 0.31 ↑G2a:ES = 0.34 ↔G2b:ES = 0.08 ↑G2c:ES = 0.50), SJ (↑G1a: ES = 0.77 ↑G1b: ES = 0.52 ↑G1c: ES = 0.96 ↔G2a:ES = 0.07 ↑G2b:ES = 0.45 ↑G2c:ES = 0.79), RSI (↑G1a: ES = 0.53 ↑G1b: ES = 0.16 ↑G1c: ES = 0.19 ↑G2a:ES = 0.27 ↔G2b:ES = 0.05 ↑G2c:ES = 0.28) G1a>G2a (MBI): 10m sprint, SJ G1a: ↑4/4 tests, G1b: ↑3/4 tests, G1c: ↑4/4 tests, G2a: ↑2/4 tests, G2b: ↑2/4 tests, G2c: ↑4/4 tests
Radnor et al. 2016 [37]	80 M	PLYO 12.7±0.3 RT 12.6± 0.3 CT 12.7±0.3 PLYO 16.4±0.2 RT 16.3±0.3 CT 16.2±0.3	UT	PHV	G1a: pre-PHV PLYO G1b: pre-PHV RT G1c: pre-PHV CT G2a: post-PHV PLYO G2b: post-PHV RT G2c: post-PHV CT	2	6	RT, PLYO, CT	time x group interaction for maturity n/a G1c>G1b (p < .05): 10m sprint (G1b:↔*1,1% G1c:↑*3,3%), max velocity (G1b:↔*0,4% G1c:↑*2,7%) G1a>G1b (p < .05): max. velocity (G1a:↑*2,8% G1b:↔*0,4%) G2a>G2b (p < .05): RSI (G2a:↑*4,6% G2b:↔*0,7%) G2b>G2a (p < .05): 10m sprint (G2a:↔*0,4% G2b:↑*1,8%), SJ (G2a:↔*1,4% G2b:↑*7,7%) G2c>G2a: 10m sprint (G2a:↔*0,4% G2c:↑*2,7%), SJ (G2a:↔*1,4% G2c:↑*12,9%) G2c>G2b: max velocity (G2b:↔*0,6% G2c:↑*3,9%), SJ (G2b:↑*7,7% G2c:↑*12,9%), RSI (G2b:↔*0,7% G2c:↑*3,8%)

Note: M, male; F, female; UT, untrained; T, trained; n/a, not available; G, group; PHV, peak height velocity; PLYO, plyometric training; RT, resistance training; CT, complex training; VJ, vertical jump; SJ, squat jump; LJ, long jump; CMJ, countermovement jump; MB, medicine ball; RSI, reactive strength index

↑, significant within-group improvement from pre to post

↔, non-significant within-group change from pre to post

MBI, interpretation based on outcomes of magnitude-based inferences

ES, effect size

#↑, substantial within-group improvements from pre to post (with >75% chance of being beneficial)

#↔, trivial change or non-substantial improvements (<75% chance of being beneficial) within-group from pre to post.

**Table 9:** Influence of the moderating variable ‘sex’ on plyometric training induced improvements in components of physical fitness.

Author	Number of subjects, sex	Age [years]	Training experience, sport-specific background	Comparator	Frequency [sessions/week]	Duration [week]	Training Type	Training effects
Skurvydas & Brazaitis 2010 [72]	23 M 13F	M 10.3±0.3; F 10.2±0.3	UT	G1: M G2: F	2	8	PLYO	groupxtime interaction n/a G1 = G2: ↑CMJ (G1: 37% G2: 38%) "G1>G2": twich torque 1Hz (↑G1: 323%, p < .001, ↔G2: 21%, p>.05)
Marta et al. 2014 [69]	37 M 43 F	M 10.7±0.4; F 10.9±0.3	UT	G1a: M, Tanner 1 G1b: F, Tanner 1 G2a: M, Tanner 2 G2b: F, Tanner 2	2	8	PLYO	G1a = G1b & G2a = G2b (p>.05): ↑MB throw 3 kg, ↑MB throw 1kg, ↑LJ, ↑VJ, ↑sprint (20m)
Steben & Steben 1981 [73]	80M 80F	n/a (7th& 8th grade), ≈12–14	n/a	G1: M, depth jump or box drills or agility/hopping/bounding G2: F, depth jump or box drills or agility/hopping/bounding	5	7	PLYO	G1>G2 (p < .05): ↑high jump, ↑tripple jump G1 = G2 (p>.05): ↑LJ
Vom Heede et al. 2007 [42]	29 M 31 F	10.6±n/a	n/a	G1a: M, RT G1b: M, mostly PLYO G2a: F, RT G2b: F, mostly PLYO	2	6	RT, PLYO	statistics n/a "G1a = G2a & G1b = G2b": ↑situp, ↑pushup, ↑pullup-hold, ↑LJ, ↑VJ, ↑MB throw (1 exception, but n/a)

Note: M, male; F, female; UT, untrained; n/a, not available; G, group; PLYO, plyometric training; RT, resistance training; VJ, vertical jump; LJ, long jump; CMJ, countermovement jump; MB, medicine ball

≈, approximately

↑, significant within-group improvement from pre to post

↔, non-significant within-group change from pre to post.

**Table 10:** Influence of the moderating variables ‘training intensity and volume’, ‘surface stability’ and ‘rest’ on plyometric training induced improvements in components of physical fitness.

Author	Number of subjects, sex	Age [years]	Training experience, sport-specific background	Comparator	Frequency [sessions/week]	Duration [week]	Training Type	Training effects
Volume								
Chaabene & Negra 2017 [74]	25 M	12.7±0.2 12.7±.0.3	UT, soccer	G1: low volume G2: high volume	2	8	PLYO	G1 = G2 (p>.05):↑5m sprint, ↑10m sprint, ↑20m sprint, ↑30m sprint, ↑CoD, ↑SJ, ↑CMJ, ↑LJ
Ramirez-Campillo et al. 2013 [75]	29 M	16.9±0.9	UT	G1: medium volume G2: medium volume hard surface G3: high volume	2	7	PLYO	timexgroup interactions n/a G1: ↓agility, ↑SJ G2: ↓CMJ, ↑DJ20, ↑DJ40, ↑5RM squat G3: ↓agility, ↑DJ20, ↓CMJ, ↑20m sprint
Chaouachi et al. 2014 [76]	42 M	13.7±0.8 13.3±0.8	UT	G1: plyo G2: plyo+balance	3	8	PLYO, BAL	timexgroup interactions n/a G2>G1 (MBI): leg-stiffness (#?G1:ES = 0.30 #↑G2:ES = 0.94ES = -0.69), 10m sprint (#?G1:ES = 0.02 #↑G2:ES = 0.86, ES = -0.57), #↑shuttle run (G1:ES = 1.21 G2:ES = 1.72, ES = -0.52) G1?G2 (MBI): #↑CMJ (G1:ES = 0.72 G2:ES = 0.88, ES = 0.17), #↑1RM leg press (G1:ES = 0.78 G2:0.68, ES = 0.14), #↑reactive strength (G1:ES = 0.59 G2:ES = 0.85, ES = 0.39), #↑30m sprint (G1:ES = 0.62 G2:ES = 0.55, ES = 0.24), #↑LJ (G1:ES = 0.79 G2:ES = 1.26, ES = 0.11), #↑star excursion balance (G1:ES = 0.64 G2:ES = 1.13, ES = 0.31), #↑stork balance (G1:ES = 0.72 G2:ES = 1.62, ES = 0.28), #↑tripple hop test (G1:ES = 1.02 G2:ES = 0.67, ES = -0.34)
Ramirez-Campillo et al. 2015c [77]	24 M	12.8±2.8 13.0±2.1	UT, soccer	G1: progressive volume G2: non-progressive volume	2	6	PLYO	G1 = G2 (p>.05): ↑vertical CMJ (G1:ES = 0.54 G2:ES = 0.23), horizontal CMJ (↑G1:ES = 0.40 ↔G2:ES = 0.13), right leg horizontal CMJ (↑G1:ES = 0.59 ↔G2:ES = 0.08), ↑left leg horizontal CMJ (G1:ES = 0.95 G2:ES = 0.36), ↑RSI20cm (G1:ES = 0.73 G2:ES = 0.23), kicking velocity (↑G1:ES = 0.34 ↔G2:ES = 0.17), 10m sprint (↑G1:ES = 0.14 ↔G2:ES = 0.16), ↑CoD (G1:ES = 0.82 G2:ES = 0.43), ↑Yo-Yo (G1:ES = 0.32 G2:ES = 0.27)
Marques et al. 2012 [78]	30 M	17.1±4.9	n/a, waterpolo	throws, workload matched: G1: heavy (3kg) G2: heavy (3kg) + light (0.4kg)	2	8	PLYO	G2>G1 (p = .004): throwing velocity water polo ball on land (G1: ↑3% G2: ↑8%) G1 = G2 (p>.05): ↑throwing velocity MB 1kg, ↑throwing velocity MB 3kg, ↑throwing velocity water polo ball in water
van den Tillaar & Marques 2013 [79]	22 M 18 F	15.9±1.0	UT	throws: G1: 3x6 (3kg) G2: 6x6 (3kg)	2	6	PLYO	G2>G1 (p = .006): throwing speed with different balls (G1:3% G2: 7%), throwing speed .35kg & 3kg (G1:<3% G2: >10%)
Intensity								
Matavulji et al. 2001 [80]	33 M	15–16	n/a, "well conditioned"	G1: 50cm height G2: 100cm height	3	6	PLYO	G1 = G2 (p>.05): CMJ (↑G1,↑G2), RFD knee extension (↑G1 ↑G2), RFD Hip extension (↔G1 ↔G2), MVC knee extension (↔G1 ↔G2), MVC hip extension,(↔G1 ↑G2)
Kobal et al. 2017 [81]	20 M	15.9 ± 1.2	n/a, elite soccer	G1: loaded jumps (+8% bwt) G2: unloaded jumps	2	6	PLYO	time x group interaction for maturity n/a G1>G2 (MBI): #↑SJ (ES = 0.43), #↑CMJ (ES = 0.33) G2>G1 (MBI): #↓sprint vel 5m (ES = -0.65), #↓sprint vel 10m (ES = -0.45), #↓sprint vel 20m (ES = -0.27) G1 = G2 (MBI): #↔MPP (ES = -0.06)
Rosas et al. 2016 [82]	63 M	12.0 ± 2.2 12.3 ± 2.3 12.1 ± 2.1	UT, soccer	G1: plyo G2: hand-held loaded plyo (+0–15% bwt) CG: soccer training	2	6	PLYO	G2>G1 (p < .05): ↑RSI (G1: 8.8% ES = 0.27 G2: 19% ES = 0.34) G1 = G2 (p>0.5): ↑right leg BJ (G1: 6.3% ES = 0.28 G2: 10.1% ES = 0.45), ↑left leg BJ (G1: 7.7% ES = 0.32 G2: 12.1% ES = 0.47), ↑BJ (G1: 6.1% ES = 0.28 G2: 7.7% ES = 0.37), ↑CMJ (G1: 4.3% ES = 0.26 G2: 7.2% ES = 0.26), ↑MKV (G1: 6.8% ES = 0.27 G2: 8.3% ES = 0.34) G2>CG (p<0.5): right leg BJ, left leg BJ, BJ, CMJ, RSI, MKV G1>CG (p < .05): RSI, MKV
Rest								
Ramirez-Campillo et al. 2014 [83]	54 M	10.4±2.0 10.4±2.3 10.3±2.3	UT, soccer	G1: 30s interset rest G2: 60s interset rest G3:120s interset rest	2	7	PLYO	G1 = G2 = G3 (p = n/a):↑CMJ (G1: ES = 0.49 G2: ES = 0.58 G3: ES = 0.55), ↑RSI 20cm (G1: ES = 0.81 G2: ES = 0.89 G3: ES = 0.86), ↑RSI 40cm (G1: ES = 0.86 G2: ES = 0.88 G3: ES = 0.98), ↑CoD (G1: ES = 1.03; G2: ES = 0.87; G3: ES = 1.04), ↑kicking distance (G1: ES = 0.39 G2: ES = 0.49 G3: ES = 0.43), ↔20m sprint (G1: ES = 0.3 G2: ES = -0.09 G3: ES = -0.13
Ramirez-Campillo et al. 2015d [84]	166 M	14.2±2.2 14.1±2.2	UT, soccer	G1: 24h rest G2: 48h rest	2	6	PLYO	G1 = G2 (p>.05): ↑SJ (G1: 4.4% G2: 3.8%), ↑CMJ (G1: 7.4% G2: 8.0%), ↑RSI20cm (G1: 12.2% G2: 12.0%), ↑LJ (G1: 5.6% G2: 5.3%), ↑20m sprint (G1: 5.6% G2: 5.1%), ↑agility (G1: 3.3% G2: 2.7%), ↑shuttle run (G1: 10.3% G2: 10.0%), ↑sit&reach (G1: 5.7% G2: 4.7%)
Surface stability								
Granacher et al. 2015 [85]	24 M	15.6±0.6 15.2±0.5	n/a, soccer	G1: stable G2: unstable	2	8	PLYO	G1>G2 (p>.01): ↑CMJ (G1: 13% G2: 4%) G1 = G2 (p>.05): ↑DJ (G1: 7.8% G2: 11.1%), ↑multiple bound (G1: 3.8% G2: .3.4%), ↑0-10m sprint (G1: 1.5% G2: 1.9%), ↔10-20m/↔20-30m/↔0-30m sprint, ↑agility figure-8 (G1:2.9% G2: 3.1%), ↑balance
Büsch et al. 2015 [86]	19 M	16.7±0.6 17.4±0.9	n/a, handball	G1: stable G2: unstable	2	10	PLYO	G1 = G2 (p>.05): ↑CMJ (G1: 3.6% G2: 8.5%, ES = 2.04), ↑SJ (G1: 11.6% G2: 4.7%, ES = 1.64), ↔DJ (G1: 11.2% G2: 5.3%, ES = 0.45), ↔broad jump (G1: 1.5% G2: 1.3%, ES = 0.72), ↔5m sprint (G1: 0% G2: 1.9%, ES = 0.74), ↑10m sprint (G1: 1.0% G2: 2.2%, ES = 1.53), ↑20m sprint (G1: 1.6% G2: 1.6%, ES = 1.59), ↑figure 8 run (G1: 2% G2: 4.5%, ES = 1.86)
Negra et al. 2017 [87]	32 M	12.7±0.2 12.2±0.5	n/a, soccer	G1: stable G2: unstable	2	8	PLYO	G2>G1 (p < .05): stable stork balance test (↔G1: 6% ES = 0.1 ↑G2: 121% ES = 1.3), unstable stork balance test (↔G1: 17% ES = .0.31 ↑G2: 149% ES = 1.83) G1 = G2 (p>.05): ↑CMJ (G1: 13% ES = 1.13 G2: 7% ES = 0.61), ↑LJ (G1: 6% ES = 1.30 G2: 6% ES = 1.41), ↑10m sprint (G1: 4% ES = 0.95 G2: 6% ES = 1.5), ↑20m sprint (G1: 4% ES = 0.74 G2: 5% ES = 1.42), 30m sprint (↔G1 ↑G2: 3% ES = 0.9), ↑agility (G1: 3% ES = 1.52 G2: 3% ES = 1.46), ↑stable Y-Balance (G1: 9% ES = 1.21 G2:12% ES = 1.49), ↑unstable Y-Balance (G1: 10% ES = 1.62 G2:19% ES = 1.50)

Note: M, male; F, female; UT, untrained; n/a, not available; G, group; PLYO, plyometric training; BAL, balance training; RM, repetition maximum; CoD, change of direction; SJ, squat jump; DJ, drop jump; LJ, long jump; BJ, broad jump; CMJ, countermovement jump; RSI, reactive strength index; MKV, maximal kicking velocity; MB, medicine ball; MVC, maximum voluntary contraction

“”, descriptive

↑, significant within-group improvement from pre to post

↔, non- significant within-group change from pre to post

↓, significant within-group impairment from pre to post

MBI, interpretation based on outcomes of magnitude-based inferences

ES, effect size

#↑, substantial within-group change from pre to post (with >75% chance of being beneficial)

#?, unclear within-group change from pre to post

**Table 11:** Influence of the moderating variable ‘training specificity’ on plyometric training induced improvements in components of physical fitness.

Author	Number of subjects, sex	Age [years]	Training experience, sport-specific background	Comparator	Frequency [sessions/week]	Duration [week]	Training Type	Training effects
Thomas et al. 2009 [88]	12 M	17.3±0.4	n/a, soccer	G1: DJ training G2: CMJ training	2	6	PLYO	G1 = G2 (p>.05): ↑VJ (G1: ES = 1.1 G2: ES = 0.7), ↑agility (G1: ES = 1.3 G2: ES = 1.5), ↔sprint speed
Steben & Steben 1981 [73]	80M 80F	n/a, 7th& 8th grade	n/a	G1:Depth Jump G2: Box Drills G3: agility/hopping/bounding	5	7	PLYO	G1>G2&G3 (p < .05): ↑high jump G2>G1&G3 (p < .05): ↑tripple jump G3>G1&G2 (p < .05): ↑LJ
Ramírez-Campillo et al. 2015 [89]	54 M	11.6±1.7 11.0±2.0 11.6±2.7	UT, soccer	G1: unilat G2: bilat G3: uni+bi	2	6	PLYO	G1 = G2 = G3 (p>.05): ↑unilateral/↑bilateral + ↑horizontal/↑vertical CMJ, ↑RSI20cm (G1:ES = 0.44 G2:ES = 0.88 G3:ES = 0.63), ↑multiple bound test (G1:ES = 0.73 G2:ES = 0.28 G3:ES = 0.64), ↑kicking velocity (G1:ES = 0.92 G2:ES = 0.26 G3:ES = 0.62), ↑15m sprint (G1:ES = 0.47 G2:ES = 0.42 G3:ES = 0.56), ↑30m sprint (G1:ES = 0.61 G2:ES = 0.31 G3: ES = 0.53), ↑agility (G1:ES = 0.8 G2:ES = 0.42 G3:ES = 0.66), ↑Yo-Yo, balance (G1:ES = 0.26 G2:ES = 0.35 G3:ES = 0.21) G1>CG: 6/21 measures G2>CG: 3/21 measures G3>CG: 13/21 measures
Ramírez-Campillo et al. 2015b [90]	40 M	11.6±1.4 11.4±1.9 11.2±2.3	UT, soccer	G1: vertical G2: horizontal G3: vert+hor	2	6	PLYO	G1 = G2 = G3 (p>.05): vertical CMJ (↑G1:ES = 075 ↔G2:ES = 0.24 ↑G3:ES = 0.51), ↑horizontal CMJ (G1:ES = 0.94 G2:ES = 0.96 G3:ES = 0.68), ↑RSI20cm (G1:ES = 0.91 G2:ES = 0.41 G3:ES = 0.62), multiple bound test (↔G1:ES = 0.53 ↑G2:ES = 0.62 ↑G3:ES = 0.63), kicking velocity (↔G1:ES = 0.47 ↔G2:ES = 0.36 ↑G3:ES = 0.67), 15m sprint (↔G1:ES = 0.49 ↑G2:ES = 0.55 ↑G3:ES = 0.99), 30m sprint (↔G1:ES = 0.30 ↑G2:ES = 0.37 ↑G3:ES = 0.63), CoD (↔G1:ES = 0.43 ↔G2:ES = 0.21 ↑G3:ES = 0.70), ↑Yo-Yo (G1: ES = 0.41 G2:ES = 0.35 G3:ES = 0.31)
McCormick et al. 2016 [91]	14 F	16.3±0.8 15.7±0.8	n/a, basketball	G1: sagital plane (linear) G2: frontal plane (lateral)	2	6	PLYO	G1>G2 (p<0.5): ↑CMJ (G1: 10.3% G2:3.8%) G2>G1 (p<0.5): ↑left LST (G1:0.6% G2:8.8%), ↑left lateral hop (G1:1.8% G2:11.9%) G1 = G2 (p>.05): ↑LJ (G1:7.9% G2:6%), ↑right lateral hop (G1:5.9% G2: 9.8%), ↑right LST (G1:3% G2:6.8%)

Note: M, male; F, female; UT, untrained; T, trained; n/a, not available; G, group; PLYO, plyometric training; CoD, change of direction; Yo-Yo, Yo-Yo intermittent recovery level 1 test; VJ, vertical jump; SJ, squat jump; LJ, long jump; CMJ, countermovement jump; DJ, drop jump; LST, lateral shuffle test; RSI, reactive strength index

↑, significant within-group improvement from pre to post

↔, non-significant within-group change from pre to post

ES = effect size

**Table 12:** Influence of the moderating variable ‘training type’ on training induced improvements in components of physical fitness.

Author	Number of subjects, sex	Age [years]	Training experience, sport-specific background	Comparator	Frequency [sessions/week]	Duration [week]	Training Type	Training effects
Radnor et al. 2016 [37]	80 M	PLYO 12.7±0.3 RT 12.6± 0.3 CT 12.7±0.3 PLYO 16.4±0.2 RT 16.3±0.3 CT 16.2±0.3	UT	G1a: pre-PHV PLYO G1b: pre-PHV RT G1c: pre-PHV CT G2a: post-PHV PLYO G2b: post-PHV RT G2c: post-PHV CT	2	6	RT, PLYO, CT	G1c>G1b (p < .05): 10m sprint (G1b:↔*1,1% G1c:↑*3,3%), max velocity (G1b:↔*0,4% G1c:↑*2,7%) G1a>G1b (p < .05): max. velocity (G1a:↑*2,8% G1b:↔*0,4%) G2a>G2b (p < .05): RSI (G2a:↑*4,6% G2b:↔*0,7%) G2b>G2a (p < .05): 10m sprint (G2a:↔*0,4% G2b:↑*1,8%), SJ (G2a:↔*1,4% G2b:↑*7,7%) G2c>G2a: 10m sprint (G2a:↔*0,4% G2c:↑*2,7%), SJ (G2a:↔*1,4% G2c:↑*12,9%) G2c>G2b: max velocity (G2b:↔*0,6% G2c:↑*3,9%), SJ (G2b:↑*7,7% G2c:↑*12,9%), RSI (G2b:↔*0,7% G2c:↑*3,8%)
Lloyd et al. 2015 [30]	80 M	PLYO 12.7±0.3 RT 12.6±0.3 CT 12.7±0.3 PLYO 16.4±0.2 RT 16.3±0.3 CT 16.2±0.3	UT	G1a: pre-PHV PLYO G1b: pre-PHV RT G1c: pre-PHV CT G2a: post-PHV PLYO G2b: post-PHV RT G2c: post-PHV CT	2	6	RT, PLYO, CT	G1a: ↑10m sprint, ↑20m sprint, ↑SJ, ↑RSI G1b: ↑10m sprint, ↑SJ, ↑RSI G1c: ↑10m sprint, ↑20m sprint, ↑SJ, ↑RSI G2a: ↑20m sprint, ↑RSI G2b: ↑10m sprint, ↑SJ G2c: ↑10m sprint, ↑20m sprint, ↑SJ, ↑RSI
Piazza et al. 2014 [92]	57 F	12.0±1.8 11.9±1.0	UT, gymnastics	G1: RT G2: low load RT + PLYO	2	6	RT, CT	G1 = G2 (p>.05): ↑CMJ (G1: 7% G2: 6.1%), ↔SJ (G1: 2.7% G2: 2.7%) G1>G2 (p < .05): ↔hopping test flight time (G1:7% G2: -5.9%) G2>G1 (p < .05): hopping test ground contact time (G1: ↔-3.6% G2: ↑22%)
Faigenbaum et al. 2007 [93]	27 M	13.6±0.6 13.4±0.9	n/a	G1: CT G2: RT+ MOB	2	6	CT, RT, MOB	G1>G2 (p < .05): shuttle run (G1:↑3.8% G2:↔0.3%), MB toss (G1: ↑14.4% G2: ↑5.6%), LJ (G1: ↑6% G2: ↔1.1%) G1 = G2 (p>.05): VJ (G1: ↑8.1% G2: ↔3.4%), ↔9.1m sprint (G1: 0.3% G2: 0.2%), ↑flexibility (G1: 27.6% G2: 29%)
Nielsen et al. 1980 [94]	381 F	7–19	n/a	G1: RT (isometric) G2: PLYO G3: sprint	3	5	RT, PLYO, SPRINT	G1>G2&G3 (p < .05): ↑MVC knee extension (G1: 33% G2: 18% G3: 16%) G2>G1&G3 (p < .05): ↑VJ (G1: 13% G2: 19% G3: 9%) G1 = G2 = G3 (p>.05): ↔10m sprint (G1: 6% G2:7% G3:2%)
Lephart et al. 2005 [95]	27 F	14.5±1.3 14.2±1.3	UT, soccer/ basketball	4 W both groups MOB + RT, then G1: MOB +BAL+ CT G2: MOB+ BAL+RT+ RT	3	(4)4	MOB, BAL, RT, CT	G1 = G2 (p>.05): leg extension ↑60°/s (G1: 8% G2: 11%) & ↑180°/s (G1: 5% G2: 15%), leg flexion ↔60°/s & ↔180°/s, ↔ hip abduction isometric peak torque
Behringer et al. 2013 [96]	36 M	15.1±1.8 15.5±0.9	n/a, tennis	G1: RT (machine) G2: PLYO	2	8	RT, PLYO	G2>G1 (p < .05): mean service velocity (G1: ↔*1,2% G2: ↑*3,8%) G1 = G2 (p>.05): ↑10RM leg press, ↑10RM chest press, ↑10RM pulldown, ↑10RM abdominal press
Vom Heede et al. 2007 [42]	29 M 31 F	10.6±n/a	n/a	G1: RT ("mENDUR", theraband) G2: PLYO/ POWER ("speed strength")	2	6	RT, PLYO, POWER	statistics n/a "G2>G1": situp, pushup, pullup-hold,LJ "G1>G2": VJ "G1 = G2": MB throw
Channell & Barfield 2008 [97]	27 M	15.9±1.2	T, football	4W technique both groups, then 8 W G1: POWER (OLY) G2: RT ("Power Lifting")	3	(4)8	POWER, RT	G1 = G2 (p>.05): ↔*VJ (G1: 4.5% G2: 2.3%) "G1>G2": VJ (ES = 0.34)
Chaouachi et al. 2014 [98]	63 M	11±1	UT, judo/ wrestling	G1: POWER (OLY) G2: PLYO G3: RT (machine-based)	2	12	POWER, PLYO, RT	time x group interactions n/a G1>G2 (MBI): #↑CMJ (ES = 0.78), #↑horizontal jump (ES = 0.63), #↑5m sprint (ES = 0.70), 20m sprint (#↑G1 #?G2, ES = 0.50) G1>G3 (MBI): #↑CMJ (ES = 0.71), #↑balance (ES = 0.60), #↑isokinetic power at 300°/s (ES = 0.44) G2>G1 (MBI): #↑isokinetic force at 60°/s (ES = 0.50), G2>G3 (MBI): #↑balance (ES = 0.86), #↑isokinetic force at 60°/s (ES = 0.54), #↑isokinetic force at 300°/s (ES = 0.48), #↑isokinetic power at 300°/s (ES = 0.47) G3>G2 (MBI): isokinetic power at 60°/s (#?G2 #↑G3, ES = 0.80), #↑5m sprint (ES = 1.2), 20m sprint (#?G2 #↑G3, ES = 0.57)
Hoyo et al. 2016 [99]	32 M	18±1 17±1 18±1	T, soccer	G1: POWER (40–60% 1RM) G2: RESP G3: PLYO	2	8	POWER, PLYO, RESP	time x group interactions n/a G1>G2 (MBI): flying 10-20m sprint (#↑G1: ES = 0.61 #?G2: ES = 0.06), #↑flying 30-50m sprint (G1: ES = 0.84 G2: ES = 0.45) G1>G3 (MBI): flying 10-20m sprint (#?G3: ES = 0.12) G1 = G2 = G3 (MBI): #↑CMJ (G1: ES = 0.51 G2: ES = 0.57 G3: ES = 0.50) G1: #↑0-50m sprint (ES = 0.60) G3: #↑flying 30-50m sprint (ES = 0.50), #↑0-50m sprint (ES = 0.46)
Escamilla et al. 2012 [100]	68 sex n/a	15.2±1.1 15.4±1.3 15.8±0.8	UT, baseball	G1: RT (throwers ten) G2: RT/POWER (pneumatic resistance) G3: PLYO	3	6	RT, POWER, PLYO	G1 = G2 = G3 (p = n/a): ↑throwing velocity (G1:1.9% G2: 1.2% G3: 2.1%)
Szymanski et al. 2007 [101]	49 M	15.3±1.2 15.4±1.1	n/a, baseball	G1: ST G2: CT(ST + MB)	3	12	RT, CT	G2>G1 (p < .01): ↑3RM dominant & ↑ 3RM non-dominant rotational strength, ↑MB hitters throw G1 = G2 (p>.05): ↑predicted 1RM squat, ↑predicted 1RM bench press

Note: M, male; F, female; UT, untrained; T, trained; n/a, not available; G, group; PLYO, plyometric training; RT, resistance training; RESP, resisted sprint training; CT, complex training; OLY, Olympic lifting; mENDUR, muscular endurance; RM, repetition maximum; VJ, vertical jump; SJ, squat jump; LJ, long jump; CMJ, countermovement jump; DJ, drop jump; CoD, change of direction; MB, medicine ball; MVC, maximum voluntary contraction; RSI, reactive strength index; ASV, average squat velocity; Yo-Yo, Yo-Yo shuttle run test

“”, descriptive

↑, significant within-group improvement from pre to post

↔, non-significant within-group change from pre to post; MBI, interpretation based on outcomes of magnitude-based inferences

ES, effect size

#↑, substantial within-group improvement from pre to post (with >75% chance of being beneficial)

#?, unclear within-group change from pre to post

*, compared to control group.

**Table 13:** Influence of alternative resistance training methods on components of physical fitness.

Author	Number of subjects, sex	Age [years]	Training experience, sport-specific background	Comparator	Frequency [sessions/week]	Duration [week]	Training Type	Training effects
Mahieu et al. 2006 [102]	21 M 12 F	12.9±1.5 11.8±1.8	UT, skiing	G1: vibration G2: body weight	3	6	VIBRA, RT	G1>G2 (p = .013): ↑high box test (G1: ES = 0.72 G2: ES = 0.37), ↑peak torque plantar flexion 30°/s (G1: ES = 0.74 G2: ES = 0.30) G1 = G2 (p>.05): ↑peak torque knee flexion 60°/s (G1: ES = 0.36 G2: ES = 0.27) & ↑180°/s (G1: ES = 0.43 G2: ES = 0.27), ↑peak torque knee extension 60°/s (G1: ES = 0.63 G2: ES = 0.35) & ↑180°/s (G1:ES = 0.63 G2: ES = 0.32), ↑peak torque plantar flexion 120°/s (G1:ES = 0.50 G2: ES = 0.41), peak torque dorsi flexion 30°/s (↑G1: ES = 0.71 ↔G2: ES = 0.51) & ↑120°/s (G1: ES = 0.68 G2: ES = 0.67)
Tous-Fajardo et al. 2016 [103]	24 M	17.0±05	UT, soccer	G1: ecc RT + VIBRA G2: "traditional"—RT + POWER + PLYO	1	11	RT, VIBRA, POWER, PLYO	time x group interaction n/a G1>G2 (MBI): CoD v-cut-test (#↑G1:5.7% ES = 1.22 #/G2:0.6% ES = 0.24), 10m sprint (#?G1:1.6% ES = 0.1 #↓G2:-5.9% ES = -1.22), 30m sprint (#?G1: -0.2% ES = -0.03 #↓G2:-6.3% ES = -0.87), jump height RJ5 (#?G1:4.2% ES = 0.23 #↓G2:-5.5% ES = -0.61), average power RJ5 (#↑G1:9.5% ES = 0.44 #/G2:-3.4% ES = -0.25) G1 = G2 (MBI): mean RSA (#/G1: 0.5% ES = 0.17 #?G2: 0.7% ES = 0.41), #?best RSA (G1: 0.3% ES = 0.08 G2: 0.7% ES = 0.27), CMJ (#/G1:4.4% ES = 0.25 #↑G2: 5.9% ES = 0.48), mean contact time (#/G1: 2.6% ES = 0.15 #?G2: 0.5% ES = 0.03), leg stiffness (#/G1: 9.3% ES = 0.25 #?G2: 1.2% ES = 0.04)
Riviere et al. 2016 [104]	16 M	17.8±0.9	T, rugby	G1: RT G2: variable RT	2	6	RT, var RT	timexgroup interactions n/a G1 = G2 (p = n/a): 1RM bench press (G1:ES = 0.2 G2: ES = 0.42), bench press velocity & bench press power at 35, 45, 65, 75, and 85% 1RM "G2>G1": relative bench press strength (G1: ES = 0.19 G2: ES = 0.41), mean velocity at 65%1RM (G1: ES = 0.36 G2: ES = 0.54) & 75%1RM (G1: ES = 0.38 G2: ES = 1.44) & 85% 1RM (G1: ES = 0.38 G2: ES = 0.86), bench press absolute mean power at 35%,45%,65% 1RM (G1: ES = 0.14, 0.10, 0.13 G2: ES = 0.27, 0.32, 0.34) & 75%, 85% 1RM (G1:ES = 0.13, 0.14 G2: ES = 0.67, 0.62), bench press relative mean power at 35%,45%,65%,75%,85% 1RM (G1: ES = 0.13, 0.07, 0.13, 0.26, 0.23 G2: ES = 0.42, 0.42, 0.55, 0.81, 0.66)

Note: M, male; F, female; UT, untrained; T, trained; n/a, not available; G, group; PLYO, plyometric training; RT, resistance training; VIBRA, whole body vibration training; VRT, variable resistance training; RM, repetition maximum; CMJ, countermovement jump; COD, change of direction; RSA, repeated sprint ability.

“”, descriptive

↑, significant within-group improvement from pre to post

MBI, interpretation based on outcomes of magnitude-based inferences

ES, effect size

#↑, substantial within-group improvement from pre to post (with >75% chance of being beneficial)

#?, unclear within-group change from pre to post

#↔, trivial change or non-substantial improvement (<75% chance of being beneficial) within-group from pre to post

#↓, within-group impairment (>25% chance of being harmful) from pre to post.

### Maturity classification

To investigate the effect of the moderator variable maturity, an adequate classification is mandatory. Since chronological age does not account for biological changes associated with maturational processes [25], different approaches have been applied to assess biological age. One of the most frequently used approaches in the literature is the Tanner staging [26] that uses secondary sex characteristics to categorize maturity levels of children and adolescents in five different stages (Tanner I-V). However, in non-clinical situations, Tanner staging might be intrusive and it does not reflect the timing of growth [27,28]. An alternative approach is the calculation of age at peak height velocity (PHV). PHV reflects the fastest upward growth in stature during puberty (growth spurt). This approach has frequently been used in recent investigations [29,30] as it represents a more reliable and practical alternative for the assessment of biological maturity [27]. To enable the comparison of studies using different methodological approaches for maturity assessment (i.e., Tanner stages or PHV-calculations), we estimated PHV using the reported chronological age. Therefore, participants aged 10–12.99 years, 13–15.99 years and 16–18 years were estimated and classified as pre-PHV, around-PHV, and post-PHV, respectively. This procedure is in accordance with Moran et al. [31]. However, these estimates need to be interpreted with caution, as the PHV is achieved over all five Tanner stages and ranges from 11.8 to 14.3 years for boys and girls [32]. For example, the application of the above mentioned method in the study of Meylan et al. [29],would have resulted in an erroneous allocation of the maturity status. Given the participants’ chronological age (14.3 years), the estimated classification would be around-PHV. However, the respective participants were categorized as post-PHV. In other words, the approach to estimate maturity status from chronological age will generate errors to an unknown degree.

## Results

**Fig 1:**
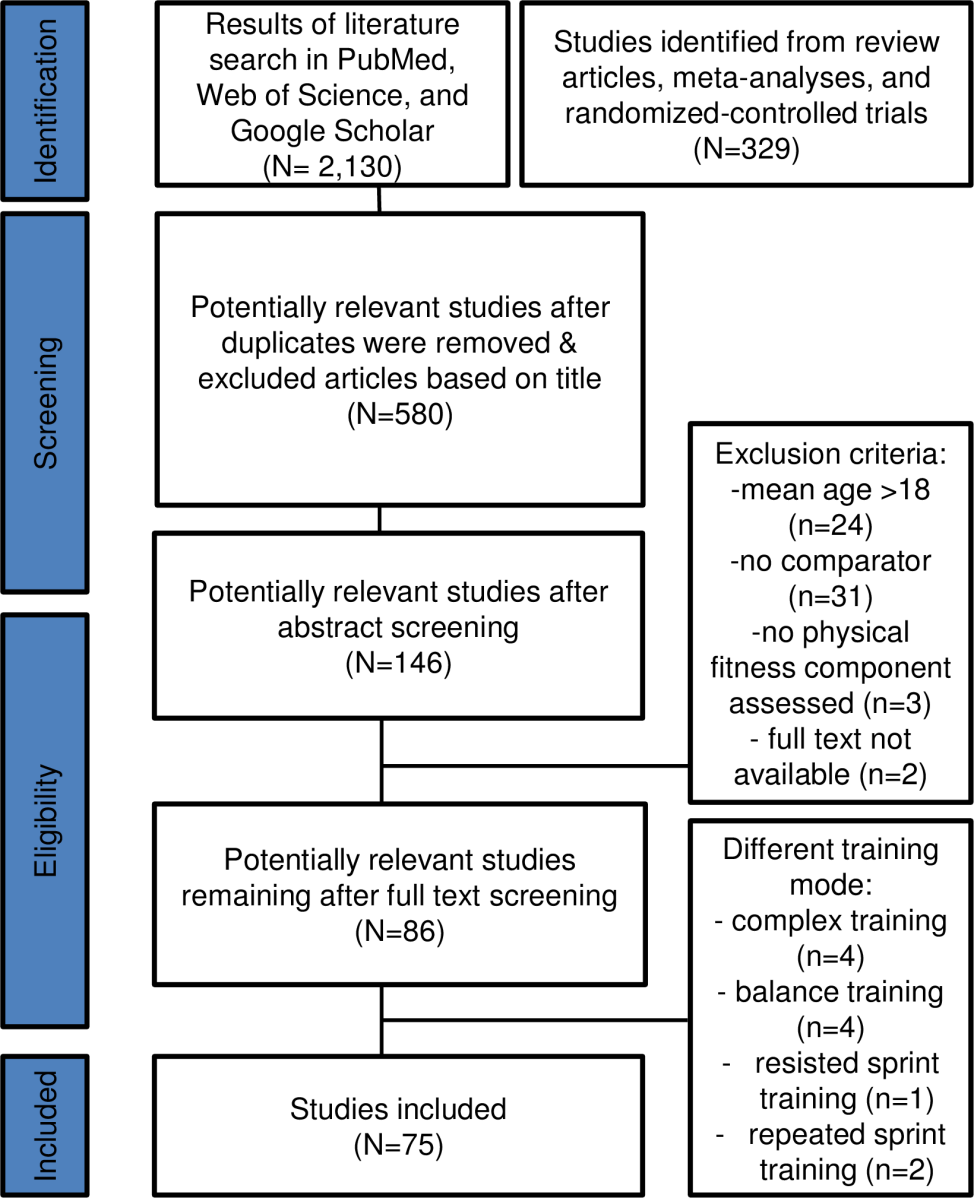
Flow chart illustrating the phases of the literature search and study selection.

Initially, our search syntax identified 2,459 potentially relevant studies (see Fig 1). Five hundred and eighty studies remained after the screening of titles. Abstract screening reduced the number to 146 potentially relevant studies. This number was brought down to 86 comparative studies after perusal of the full texts. Another 11 comparative studies were excluded because they did not apply either single-mode resistance or plyometric training (Fig 1). Finally, 75 comparative studies were eligible for inclusion in this literature review. Overall, 5,138 participants (24% female, 76% male) were enrolled in the identified studies. On average, duration of resistance and plyometric training lasted 8.9 ± 3.6 weeks and 7.1±1.4 weeks, respectively. Both, the influence of maturation (resistance training: 9 studies, plyometric training: 5 studies) and the influence of sex (resistance training: 8 studies, plyometric training: 4 studies) on resistance and plyometric training-related effects on components of physical fitness in youth have been examined (resistance training: Tables 1 and 2, plyometric training: Tables 8 and 9). Forty studies investigated the effects of training descriptors (e.g., intensity and volume, periodization) on physical fitness outcomes (e.g., muscle strength [1RM] and power [jump height]). Tables 4–6 illustrate training descriptors for resistance training, Tables 10 and 11 for plyometric. Another 13 studies examined how training type influenced physical fitness outcomes (resistance training, plyometric training, complex training, see Table 12). Additionally, three studies each were identified that investigated the influence of alternative resistance training methods (Table 13) or supervision (Table 7) on outcomes of physical fitness in youth. The explanation for these numbers exceeding the total of 75 studies is due to inclusion of some studies (e.g.,[33]) to perform multiple sub-analyses (e.g., maturity and sex).

### Resistance training

#### How maturation influences resistance training induced adaptations

In the past, it has been discussed intensively whether trainability of physical fitness changes with maturation of children and adolescents [105,106]. Especially the growth-related changes in concentrations of circulating hormones were thought to be crucial for training induced physiological adaptations [107]. Nine comparative studies were included in our analysis that specifically examined the influence of maturity or chronological age on adaptations following resistance training. For instance, Lillegard et al. investigated the effects of a 12 week resistance training program on either pre- or post-pubertal boys and girls and found no maturation-related effects on gains in 10 RM strength, irrespective of participants’ sex [33]. Of note, self-assessment of Tanner stages, as partly executed in this study, appears not to be an adequate means [108] which is why results have to be interpreted with caution. In another study, Pfeiffer and Francis found that prepubescent boys had greater relative improvements in three out of 16 strength tests (torque/kg body weight at 30°/s or 120°/s) compared to mid-pubescent and post-pubescent youth after nine weeks of resistance training [36]. In contrast, Vrijens reported that pre-pubescents improved their lower/upper limb strength (maximum voluntary contraction [MVC] arm/leg flexion/extension) after an eight week resistance training program, while trunk flexion and trunk extension strength (MVC) increased in both, pre- and post-pubescent boys [4]. Steinmann reported larger improvements in some (e.g., lower body power [vertical jump height], strength [1RM bench press, 1RM squat]) but not all fitness outcomes (e.g., speed, upper body power [medicine ball throw]) for (estimated) around-PHV compared to pre-PHV children [35]. Gabbett et al. detected mixed results when comparing (estimated) around-PHV and post-PHV cohorts [34]. While around-PHVs showed larger increases in upper body muscular endurance (chin-up repetitions in 60 seconds), post-PHVs showed larger improvements in lower-limb muscular endurance (multi- effort vertical jumps). Meylan et al. examined the effects of resistance training in pre-, around- and post-PHV youth on measures of muscular strength, power and speed. They found larger training-related effects on muscular power (estimated from the power–load relationship), maximum strength (1RM) and speed (e.g. 10m sprint) in youth estimated around -and post-PHV compared to pre-PHV children [29]. Also Moran et al. found an eight week resistance training to improve lower body isometric strength (mid-thigh pull) in post-PHV swimmers to a greater extent than in pre-PHV subjects [38]. Of note, vertical jump height (despite smaller within-group effect sizes) showed greater improvements in the pre-PHV cohort (in comparison to the maturation-matched control groups). In contrast, Lloyd et al. did not detect any differences in speed (e.g. 10m sprint) or power performances (e.g. squat jump height) after resistance training with pre- compared to post-PHV cohorts [30].

Table 1 categorises the abovementioned studies and their training effects. Taken together, comparative studies suggest that the overall trainability of physical fitness following resistance training appears to produce mitigated effects during pre-PHV with no clear differences between around- and post-PHV youth. A previously conducted meta-analysis partly supports these findings by showing that the trainability of muscular strength only slightly increases with age [6]. This increased resistance training response was also observed in a recent meta-analyses conducted with adolescent females aged >15 compared with females aged <15 [109]. While strength gains in pre-pubertal children occur mostly due to neural adaptations [110], additional morphological adaptations, due to the increase of sex hormones with the onset of puberty, likely explain the increased effects of resistance training in the later stages of maturation [12]. In contrast, even though absolute strength gains are smaller in pre-PHV cohorts, relative strength gains are comparable and can even be larger compared to around and post-PHV cohorts when bodyweight is taken into account [4,35,36,111,112]. Most studies were conducted in male youth, hardly any in females. Only one study [33] included females. However, this study did not investigate the post-PHV period, only around and pre PHV. Thus, there is no conclusive evidence from comparative studies on the effects of maturation on resistance training induced strength gains in girls.

#### How sex influences resistance training induced adaptations

It is well-documented that during maturation, natural strength development largely differs between boys and girls [12,113]. During pre-puberty, strength gains are reported to be similar between sexes. With the onset of puberty, primarily boys experience tremendous strength increases. It has been argued that these strength gains are due to hormonal changes that occur during puberty [25]. However, sex-related differences in strength development are sometimes misinterpreted as differences in strength trainability. It is important though that maturity related strength development and the trainability of strength are two different entities and should for that reason be interpreted independently. Therefore, the following paragraphs focus on sex-specific effects of resistance training on measures of physical fitness. Notably, a previous review [114] concluded that in adults, relative training-related strength increases are similar between men and women if the same exercise stimulus is delivered.

Our systematic literature search identified eight studies that examined the effects of the moderator variable sex on resistance training-related physical fitness outcomes. For instance, Vom Heede et al. reported similar gains (with one exception) in several strength and power outcomes (e.g., pull-up-hold, jump distance/height, med ball throw) for both sexes (age 10.6 years) after resistance training, which focused on the development of muscular endurance or speed strength [42]. Unfortunately, inferential statistics and the single neuromuscular outcome that differed between the groups were not reported in this study, which is a limiting factor when it comes to the interpretation of study findings. Similarly, Meinhardt et al. and Benson et al. looked at the effects of resistance training in 12 year olds and did not find any differences in the change of maximum strength (1RM) between girls and boys for muscles of the lower and upper body (leg press and smith press and bench press ad leg press, respectively) [40,41]. Unfortunately, the experimental groups in the study of Meinhardt et al. [40] differed in their maturation status with the majority of boys being pre-pubertal and the majority of girls being post-pubertal, which impedes a direct between-sex comparison. Additionally, Siegel et al. did not detect any differences in trainability of muscle strength (chin-up repetitions) and muscle endurance (flexed arm hang) between 8-year old boys and girls after a 12-week resistance training [39]. Similarly and in line with these findings, Hassan as well as Letzelter and Diekmann reported no sex-related differences in maximal strength (static and dynamic relative force of knee extension and 1RM bench press/ 1RM squat, respectively) gains between pre-pubertal boys and girls following 6-weeks or 12- weeks of resistance training, respectively [43,45].

Controversial results were observed by Muehlbauer et al., who studied the effects of high velocity resistance training on proxies of strength and power in (estimated) post-PHV boys and girls [44]. In terms of jump height, no significant differences were observed in training-related improvements between sexes. However, isometric maximal strength and rate of force development of the leg extensors increased to a larger extent in girls compared with boys. In contrast Lillegard et al. reported larger training-related improvements of 10 RM strength (2 out of 6 exercises) for boys in 10 year- and 13.3 year-old cohorts [33].

In summary, the majority of the identified studies indicate that resistance training induced adaptations are similar between males and females of the same age group [39–43,45]. These results are partly in line with findings from a recently published systematic review and meta-analysis [23]. These authors revealed that boys and girls adapt similarly to resistance training protocols regarding their strength and jump performances. Nevertheless, it is important to note that only two studies in the present systematic review matched their subjects for maturation [33,44]. As was discussed previously (see section methods: maturity classification), this assessment might not be accurate enough and therefore not adequate to estimate the respective growth and maturation status. Given that resistance training appears to have the largest effects in around- and post-PHV cohorts (see section: maturation and resistance training induced adaptations), and that comparative studies taking maturation and sex into account are scarce [33,44], future research should focus on the role of sex and maturity in resistance training studies with a focus on around- and post-PHV youth.

#### Effect of training descriptors on resistance training induced physical fitness outcomes

Training descriptors that specify the exercise stimulus are known to have a significant effect on specific physical fitness outcomes [1]. Therefore, it is recommended to define those descriptors as precisely as possible to allow an adequate interpretation of the training induced effects [115]. Moreover and with reference to comparative studies, only one descriptor should vary between groups so that differences in adaptations can be attributed to this specific parameter.

#### How intensity and volume influence resistance training induced adaptations

It has previously been reported that low-intensity high-repetition protocols [48] produce larger strength gains (e.g. significant increases in 1RM chest press compared to control the group) in untrained children when compared to high-intensity low-repetition protocols (no difference to the control group). It was argued that in inexperienced children, it appears to be important to execute sufficient repetitions per set to elicit such strength gains [48]. This assumption is supported by later findings presented by Faigenbaum et al. showing that upper body strength (1RM chest press) of children significantly increased (compared to the control group) after a low-intensity high-repetition protocol, but not after a high-intensity low-repetition protocol [47]. Further, if explosive medicine ball passes were added to the high-intensity low-repetition protocol the improvements in chest press strength (1RM) were comparable with the low-intensity high-repetition group. By contrast, a follow-up study from Faigenbaum et al. and another study by Steele et al. found no training-related differences in strength gains (e.g. 1RM chest press/ 15RM leg press and 1RM bench press/ muscular strength endurance bench press, respectively) with high intensities and low repetitions versus training with lower intensities and higher repetitions [49,51]. Surprisingly, in an early study, Rarick and Larsen did not even detect a difference in maximal static/isometric strength when performing daily low intensity and low volume compared to high intensity and high volume training for the wrist flexors in male adolescents for four weeks [116]. However, the training duration of this study was rather short which is why the results have to be interpreted with caution.

While the majority of the above cited studies [47,49,51] examined untrained children, Gonzalez-Badillo et al. looked at strength gains in 16-year-old well-trained weight lifters [52]. These authors observed that a high volume protocol over ten weeks did not induce greater adaptations (e.g. 1RM squat/ clean and jerk) than a low volume protocol when relative intensities were kept constant among groups. Of note, a medium volume approach with less than 85% of the maximally tolerated volume elicited the greatest gains in the 1RM snatch. A second study by Gonzalez-Badillo et al. conducted on with junior weightlifters investigated how different distributions of relative intensities (low/medium/high amount of training intensities >90% 1RM) have an impact on training-related fitness outcomes when the total training volume and frequency were kept constant [53]. Again, strength gains (e.g. 1RM clean and jerk) were greatest with a program that applied medium training volume of high training intensities. No significant differences in strength gains were found if low volume of high intensity training was contrasted with high volume of high intensity training. In terms of training volume, Yuktasir & Tuncel compared a protocol consisting of three sets per exercise with a single set training in 16 to 17-year-old boys [50]. While the three sets were performed until concentric muscle failure ensued, the single set training provided manual resistance (for the eccentric part) and assistance (for the concentric part) allowing for further repetitions after concentric muscle failure occurred. The single set was performed until the eccentric phase was no longer durable. Strength gains (1RM and MVC) were comparable between both protocols.

Of the above-cited studies, four examined the effects of resistance training on measures of muscular endurance. Two studies conducted by Faigenbaum and co-workers [47,48] showed larger effects on muscular endurance (e.g. number of chest press repetition with pre training 1RM) if more repetitions during a single set to muscle failure were performed in children. Another study by Faigenbaum et al. contrasted a single set with either medium (6–10 repetition) or high volume (15–20 repetition) to muscular failure in boys and girls [49]. Even though no group differences for muscular endurance gains (15RM leg press) were observed, only the higher repetition protocol resulted in larger improvements compared to the control group, thus favouring higher repetitions [49]. In contrast, Steele et al. did not observe protocol specific effects on muscular endurance (bench press repetitions with 70% of 1RM) when training was conducted with two sets of either low (4–6) or high (12–15) repetitions to momentary failure in adolescents [51]. They argued that there appears to be a minimum threshold for both volume and intensity which is necessary to optimize adaptations. This threshold could be reached when performing a second set. Interestingly, the inclusion of 6–8 medicine ball throws to a single set of a high load and low repetition protocol resulted in comparable gains in muscle endurance (bench press repetitions with pre-training 1RM) compared to the medium load and high repetition protocol in children [47]. This finding supports the minimum threshold hypothesis.

In summary, the identified studies that examined the effects of different intensities, repetitions and volumes in resistance training with youth show conflicting results. Thus, there is no conclusive evidence from comparative studies in terms of optimal intensity-volume relations that are needed to improve maximal strength in youth. Interestingly, there is currently no comparative study available that observed larger training-related improvements with high intensities compared to low intensities in youth. This is contrary to the results of a recent meta-analysis[23]. These authors observed that intensities (>80% 1RM) in combination with multiple sets (5 sets) are most effective to improve muscle strength in young athletes. The observed differences in findings between our results from comparative studies and the meta-analysis can most likely be explained by differences in the study duration (8.1±1.8 weeks in comparative studies versus >23 weeks in the meta-analysis), training volume (mostly 1 set in comparative studies versus 5 sets in the meta-analysis), and the examined cohorts (untrained subjects in comparative studies versus young athletes in the meta-analysis). Moreover, the work of Gonzalez-Badillo et al. in trained youth indicates that an often cited statement by coaches “more is better” might not hold true if the goal is to induce optimal performance gains[52,53]. However, further studies are needed to confirm these findings. For the development of muscle endurance, comparative studies suggest that higher repetitions are superior, at least if a single set is performed. Future comparative studies should also specifically compare trained with untrained subjects.

#### How rest intervals influence resistance training induced adaptations

It has previously been reported that children and adolescents show faster recovery between sets of resistance training [117] and may even resist fatigue to a greater extend [1,118] compared with adults. Additionally, differences have also been observed in fatigue resistance between children and adolescents, with children showing an improved ability to withstand the drop in peak torque during multiple sets of resistance training [119]. It seems plausible to argue that these acute differences within youth populations may also have an impact on long-term training adaptations. However, to date there is no study available that examines the influence of inter-set and inter-day rest- intervals during long-term resistance training in youth using comparative studies.

#### How training frequency influences resistance training induced adaptations

From adult research, it is known that training frequency plays an important role for the design of resistance training programs. For instance, training twice per week is superior to training once per week to induce muscle hypertrophy [120]. In addition, a review by Tan concluded that 3–5 sessions per week seem to be the optimal frequency for adults to maximise strength gains [121].

In youth, the role of training frequency is less clear. Out of six identified studies that examined training frequencies, four compared one versus two training sessions per week. Three of the identified studies reported larger gains in physical fitness (e.g. strength, power, speed) in children and adolescents for two compared to one training session per week [35,54,55]. Unfortunately, the applied statistical analyses were insufficient, non-inferential, or not applicable. A study by DeRenne et al. examined the effects of resistance training frequency on the ability to maintain strength [56]. After a 12 week pre-season training program with three sessions per week, pubescent baseball players followed a resistance training protocol either once or twice per week for another 12-weeks during the baseball season. No significant between-group differences were found over time in 1RM strength (e.g., leg press) or muscular endurance (pull-up repetitions). To the authors’ knowledge, only one study compared higher training frequencies in youth. Uppal and Tunidau contrasted the effects of two, three, and five training sessions per week and found significant larger improvements for muscular endurance (pullup/sit up repetitions) and power performance (broad jump distance) after the training with five sessions when compared with two training sessions per week [57]. Notably, no statistically significant differences were found between the groups that performed five and three and two and three training sessions per week.

Taken together, two resistance training sessions per week appear to produce larger training-related gains in physical fitness (e.g. maximal strength, muscular endurance) than one session per week, particularly in untrained youth. However, further research is needed to investigate if more than two sessions produce additional effects. Unfortunately, all of the identified studies did not report the maturational status of the enrolled subjects which is why results have to be interpreted with caution.

#### How periodization influences resistance training induced adaptations

Another important training descriptor in resistance training is the application of appropriate and performance enhancing periodization models. This has previously been shown in a meta-analysis on the effects of periodization in strength and power training on measures of muscle strength and power in adults. The study revealed that periodized training programs are more effective than non-periodized programs, irrespective of sex, training background, or chronological age [122]. However, findings from a recently published literature review indicate that periodized versus non-periodized resistance training programs do not produce extra effects on measures of muscle size and muscle strength [123]. To our knowledge, only one comparative study tested the effects of periodization on maximal strength (and flexibility) in youth. In this study, a 12-week non-periodized resistance training program was compared to a nonlinear periodized resistance training program [58]. Both programs produced similar effects in maximal strength (1RM). Of note, the nonlinear periodization program resulted in greater effect sizes than the non-periodized program and might, therefore, be superior. Different periodization models were applied in adult research and it was found that linear and undulating periodization models show similar strength gains [124]. In youth, research on the effects of periodized training programs is scarce. The available three studies that compared linear with undulating periodization are in line with the reported findings on adult research. That is, the different types of periodization produced similar strength gains in obese [61], sub-elite [59], or elite young athletes [60].

#### How does the exercise mode and type influence resistance training induced adaptations

Four studies examined the impact of different resistance training modes (e.g., isometric, isotonic) and forms (e.g., machine-based, free weight) on measures of muscle strength in youth. In terms of exercise mode, Shields et al. compared an eight week resistance training, that was either conducted in isokinetic or isotonic mode, on maximal isometric leg strength and muscle endurance in adolescents [62]. Similar improvements were found for leg strength (MVC) and muscular endurance (isotonic leg flexion/ extension endurance test). Of note, isokinetic training led to better results in isokinetic leg press strength at 30° per second. In another study, Smith and Melton found similar effects of an isotonic and either a slow or fast isokinetic resistance training program on measures of leg strength (isometric, isotonic and isokinetic) in male adolescents [63]. In contrast, the applied isokinetic training at fast movement velocities improved jump and sprint performances (e.g. vertical jump height, 40 yard time), to a larger extent compared with isokinetic training at slow movement velocities and isotonic training. This study is methodologically limited in as much as no inferential statistics were applied.

In terms of exercise type, Bulgakova et al. examined the effects of a machine-based resistance training program versus a sport-specific conditioning program (tethered swimming) in (estimated) pre-PHV swimmers [64]. It was found that machine-based training improved measures of muscle strength (e.g., dry land strength endurance) more than a sport specific conditioning program. However, swimming technique suffered from machine-based training while it was promoted through sport-specific training.

Flanagan and co-workers compared machine-based resistance training to resistance training that was conducted with the own body weight in untrained male and female children [65]. They observed larger improvements for the upper body power performance (medicine ball chest pass) after bodyweight training, whereas neither group improved in speed/ change of direction performance (shuttle-run) or their lower body power performance (long jump distance). However, between group baseline differences were found for the medicine ball throw test. Since the authors did not adjust their analyses for baseline differences, findings have to be interpreted with caution.

From the above mentioned studies, it is evident that different training types (e.g., bodyweight, machine) are capable of improving measures of strength or power in youth. It appears that the type of exercise mode (e.g., isotonic, isometric) does not influence strength gains in youth. Since adult research has shown that eccentric contraction seem to elicit greater strength adaptations than concentric contractions [125], eccentric exercises (e.g., Nordic hamstring exercise) have been safely incorporated in youth injury prevention programs [126], and no studies in youth population compared these contraction types, future research should investigate this exercise mode in youth.

#### How supervision influences resistance training induced adaptations

Supervision during youth resistance training is important to ensure appropriate exercise technique and to avoid injuries [1]. There is additional evidence from comparative studies to suggest that supervision may also increase the efficacy of youth training programs. In fact Coutts et al. and Smart and Gill observed that supervised versus non-supervised resistance (and speed and endurance) training resulted in greater strength gains (e.g., 3RM and 1RM, respectively) [66,68] and better speed and power performances (10m sprint and vertical jump height) [68] in adolescents rugby players. It has to be noted though that adherence rates were higher in the supervised group which may have influenced the findings. In contrast, when supervision was compared to an online-video-based training program, similar effects were observed for measures of power and speed (vertical jump height and 20m sprint) in male and female youth [67]. However, supervision had an effect on “movement quality” as assessed with the functional movement screen. Interestingly and similar to the study of Coutts et al. [66], adherence was greater in the supervised (97%) compared with the non-supervised group (77%). Thus, supervision may affect training-induced outcomes through higher adherence rates and better instructions, other factors might also contribute to the observed divergent outcomes (e.g., training intensity, motivation).

### Plyometric training

#### How maturation influences plyometric training induced adaptations

The literature search revealed a total of five studies that examined the effects of plyometric training in general and the influence of the moderator variable maturation in specific on physical fitness outcomes. For instance, Moran et al. compared a 6-week plyometric training with either pre- or around-PHV boys and detected larger training-induced improvements in 10-m sprint time for the around PHV-group [70]. In contrast, Lloyd et al. observed that plyometric training resulted in improved reactive strength index (RSI) and leg stiffness in youth soccer players in the estimated “late” pre-PHV (12.3±0.3 years) but not in the “early” pre-PHV (9.4±0.5 years) cohort [71]. Of note, around-PHV (15-year) peers only improved their leg stiffness. Marta et al. scrutinized the trainability of lower and upper body plyometric exercises (and endurance) adaptations in Tanner stage one compared to two (estimated pre-PHV) boys and girls and did not detect any between group differences over time in the examined outcome measures (e.g., medicine ball throw, long jump distance, 20m sprint) [69]. It has to be noted though that in this study Tanner stages were self-assessed which is why the maturation classification and the corresponding results might be inaccurate. Another study by Lloyd and co-workers found plyometric training to be more effective in pre- compared to post-PHV individuals, indicated by greater improvements in squat jump and 10m sprint performance [30]. Radnor et al. examined the effects of either strength, plyometric, or complex training in pre- and post-PHV subjects [37]. Even though outcomes were only compared between training types, but not between pre- and post-PHV cohorts, they found that plyometric training produced larger effects on maximal running velocity in the pre- PHV cohort compared to resistance training. In contrast, training effectiveness was lower for plyometrics compared to resistance training for the ability to accelerate (10m sprint) and squat jump in the post-PHV group. Therefore, if plyometric training is realized as a single training regime, it seems to induce inferior results in the post-PHV cohort compared to resistance training.

Taken together, the results of the above mentioned studies indicate a better trainability of plyometrics in the pre-PHV phase. This is partly in line with a recent meta-analysis reporting that plyometric training is moderately effective during the pre- and post PHV periods and less effective during the around-PHV period in improving countermovement jump performance [10]. Interestingly, a meta-analysis that examined the influence of maturation on plyometric training related effects on change of direction (COD) performance observed a tendency towards larger improvements for around- and post-PHV individuals compared to pre-PHV children [127]. Unfortunately, none of the above mentioned comparative studies investigated the effects of resistance training on COD tasks. The underlying reasons for the divergent outcomes between the effects of plyometrics on jump and COD performances in pre- and around-PHV individuals are unclear, but the technical component needed for COD movements [128] might play a role. In addition, comparative studies [30,37] in pre-PHV compared to post-PHV youth indicated larger improvements for 10m sprint and jump performances. This is in contrast to the results of a recent meta-analysis [31]. Of note, Moran et al. observed moderate effects of plyometric training on countermovement jump performance in both, pre- and post PHV youth. It has been speculated that the inferior results of plyometric training in post-PHV cohorts might be due to a possible inability to adequately increase concentric strength [37], which is an important factor for accelerating the body and squat jump performance [129,130]. Additionally, an increase in neural coordination and central nervous system maturation [131] in pre-PHV children may be responsible for the observed larger effects of plyometric training in this period and has recently been described as “synergistic adaptation”, that is the simultaneous adaptation to specific (training induced) and natural (growth and maturation) processes [30]. Of note, older children within the pre-PHV period might adapt to a larger magnitude (RSI, leg stiffness) to plyometric training compared to their younger counterparts [71]. Finally, future studies should include measures of COD performance, compare around- and post-PHV groups, clarify whether within pre-PHV differences exists and have a clear classification of maturity and the applied training stimulus.

#### How sex influences plyometric training induced adaptations

To date, four studies examined sex-related effects following plyometric training. Three studies investigated (estimated) pre-PHV children [42,69,72] and one study investigated (estimated) late pre-PHV and/or around-PHV children/ adolecents. The study of Skurvydas and Brazaitis did not detect any training-related differences between sexes in countermovement jump (CMJ) height and muscle thickness after eight weeks of plyometric training [72]. However, these authors observed larger effects for twitch torque in favour of boys. In contrast Vom Heede et al. did not find divergent outcomes (with one exception) in several strength and power outcomes (e.g., pullup-hold, long jump distance, med ball throw) between boys and girls after a primarily plyometric training [42]. In addition to the before mentioned limitations (see section sex and resistance training) of this study, two out of seven exercises were resistance training exercises (for the upper and lower back), thus making conclusions even more uncertain. Also Marta et al. compared the effects of plyometric training among male and female children [69]. These authors could not detect any effects of the moderator variable sex on training-induced jump, sprint and medicine ball throw enhancements, irrespective of the examined maturity status. For estimated late pre-PHV and/ or around-PHV peers (approximately 12–14 years), Steben and Steben reported larger improvements for boys compared with girls in high- and triple jump performances after plyometric training [73]. Unfortunately, these authors did not report either mean age or maturity status.

Taken together, the trainability of plyometrics in pre-PHV children appears not to be affected by sex-specific effects, with the exception of one tested parameter (twitch torque) in one study [72]. In contrast, a single study in youth of estimated late pre-PHV or around-PHV found larger improvements in jump performances for males compared with females, although due to major study limitations, this has to be viewed with caution (73). The possible physiological reasons for this observation are unclear. Several factors associated with the menstrual cycle in women, such as altered recovery [132] or changes in strength [133,134] might affect measures of physical fitness. This especially holds true when pre-post testings are performed in different phases of the menstrual cycle due to study duration.

Anyhow, since three out of four studies show major limitations, conclusions for the general youth population are not possible. Additionally, post-PHV boys and girls have not been examined yet. Since sensitivity to plyometric training programs may change differently according to sex over time/maturation, future studies should elucidate sex-specific adaptational differences following plyometric training in pre-, around-, and post-PHV cohorts separately. Also, the possible influence of the menstrual cycle needs to be taken into account when investigating around- and post-PHV females.

### Effect of training descriptors on plyometric training induced physical fitness outcomes

#### How intensity and volume influence plyometric training induced adaptations

Comparative studies on plyometric training volume show diverse results. A recent study compared protocols with either low (50–60 contacts per session) or high volume (110–120 contacts per session) and found that both protocols resulted in similar increases in measures of speed and power (10m sprint and CMJ) in prepubertal soccer players [74]. Ramírez-Campillo et al. examined the impact of a seven-week plyometric training program with either medium training volume on soft (athletic mat) or hard (wooden gym floor) training surface (780 drop jumps total, 60 per session) or high training volume on soft surface (1560 drop jumps total, 120 per session) [75]. They found that the protocol with high training volume on soft surface elicited more pronounced performance improvements (e.g., 20 cm drop jump, 20m sprint) compared to the medium volume training protocol (e.g., squat jump) on soft surface in untrained male adolescents. Of note, the medium training volume on hard surface also resulted in larger performance improvements in the 20 cm and 40 cm drop jump tests and the 5-RM squat test which is indicative of a time efficient training program. A study carried out by Chaouachi and co-workers contrasted a plyometric training program with a plyometric training that substituted 50% of the plyometric training volume with balance training exercises in untrained male adolescents. [76]. They found larger improvements in components of physical fitness (e.g. speed, power) for the plyometric plus balance group compared with the plyometric training only group. This suggests that a lower plyometric training volume (50% vs 100%) might be as effective. Interestingly, increasing the training volume over time appeared not to elicit statistically different fitness outcomes in youth soccer players (age 12.8± 2.8 years and 13.0±2.1 years) compared to no progression in training volume. However, larger practically meaningful improvements were observed for maximal kicking velocity and 10m sprint performance [77].

With regards to plyometric training for the upper body, Marques et al. compared the effects of a training program using throwing exercises conducted in the stretch shortening cycle [78]. While one experimental group exercised with heavy balls (3 kg), the other group exercised with a combination of heavy balls and water polo balls (approximately 0.4 kg). The total workload between groups was matched. Both groups showed similar improvements in throwing velocity outcomes which is why the authors concluded that workload might be a critical factor for increasing throwing velocity. This is further supported by findings from Van den Tillaar et al. who reported that higher training volume with same intensities elicited larger improvements in throwing speed with different balls than lower training volume in adolescent athletes [79].

Different intensities in plyometric training have recently been investigated [81]. Elite soccer players (estimated post-PHV) exercised twice per week for 6 weeks with unloaded or loaded (additional weights equalling eight percent of their body weight) vertical and horizontal jumps. Greater improvements in jump performance (e.g., CMJ) were observed for the loaded condition, whereas the decline in sprint velocity in both groups was less distinct for the unloaded condition. Rosas et al. compared an unloaded with a loaded plyometric training (hand-held weights) in youth soccer players of ages 10–16 [82]. They found larger performance improvements after 6 weeks due to added load (0–15% of body weight) compared to regular plyometric training only for the RSI, albeit the loaded intervention was superior to the control group in all seven performance tests, whereas the unloaded plyometrics was superior only in two tests. Matavulj et al. examined the influence of different intensities by comparing different drop heights (50 cm versus 100 cm) while performing drop jumps in well-trained adolescent basketball players and found similar improvements in muscular power (CMJ height, rate of force development knee/ hip extension) and strength (MVC knee/ hip extension) in both groups [80].

Taken together, the above mentioned studies suggest that low-to-moderate volume of lower body plyometric training might be sufficient and more time efficient to induce improvements in physical fitness (e.g., power, speed) compared to high volume training. However, training surface (i.e., soft versus hard surfaces) has to be taken into account and might affect training-related outcomes as well. Studies with adults have shown that plyometric exercises performed under unstable conditions can result in performance decrements [135] and altered kinematic responses (joint range of movement) compared to plyometrics under stable conditions [136,137]. Research comparing plyometrics on stable versus unstable surface in children and adolescents showed mixed results [85–87]. While Negra et al. observed positive effects of unstable plyometric versus stable plyometric training in pre-PHV boys on measures of static balance, they could not find an effect on jump performance, change of direction speed or dynamic balance [87]. Further, Büsch et al. were not able to detect differences in jump and sprint performances after ten weeks of plyometric training on stable or unstable surfaces in adolescent handball players [86]. In contrast, Granacher et al. detected larger jump performance (CMJ) improvements in male adolescent soccer athletes after training on stable surfaces, while speed, change of direction performance or balance did not differ between the stable and unstable groups [85].

Plyometric training intensities can be modified in different ways (e.g., extra load, drop height). Additional training loads seem to be beneficial [81,82], whereas an increase in drop height was not [80]. Of note, to the authors’ knowledge, no studies exists that compared the effect of added loads between plyometrically untrained and trained cohorts, albeit these effects might be training status dependent. Therefore, future comparative studies need to elucidate the impact of volume and intensity in plyometric training on physical fitness by examining the influence of training expertise (e.g., untrained, well-trained, and plyometrically trained). Also and in accordance with Ramirez-Campillo et al. the training surface should be reported to allow for better comparison of study outcomes [19].

#### How rest intervals influence plyometric training induced adaptations

Regarding plyometric training in the adult population, data for optimal rest periods between plyometric sets do not exist, but an inter-day rest of 48 to 72 hours was previously recommended [138]. Interestingly attenuated symptoms of exercise-induced muscle damage have been observed in children compared to adults after plyometric training [139]. Therefore, Ramírez-Campillo et al. investigated the effects of inter-day [84] and inter-set [83] rest on power and speed (e.g. 20m sprint, CMJ) in male adolescents and children, respectively. Findings from these studies show that neither the amount of inter-set (30 s, 60 s or 120 s), nor inter-day rest (24 h or 48 h) affected plyometric training outcomes differently. Since the response to the same plyometric training stimuli seem to differ during maturation (see section maturity), it seems plausible that differences in interday and interset rest might also be adequate means to optimize plyometric training outcomes in pre-, around- and post-PHV youth. Therefore, future comparative studies should explicitly examine the influence of sex and maturation.

#### How training frequency influences plyometric training induced adaptations

Plyometric training in youth populations has been shown to be effective with different training frequencies (e.g., two or three sessions per week, see Table 5).

With regard to adult populations, plyometric training has been found to be equally effective with a moderate frequency (and volume) of two weekly sessions compared to four weekly sessions [140]. Unfortunately and to the authors’ knowledge, no studies have been conducted comparing different plyometric training frequencies in children and adolescents. Therefore, there is an urgent need for researchers in the field of youth strength and conditioning to clarify what frequencies of plyometric training are adequate and effective (including untrained, athletes, male and female, as well as the different maturation stages) in youth.

#### How the principle of training specificity affects plyometric training induced adaptations

Plyometric training programs vary in several parameters, such as exercise selection and execution. Thomas et al. found the jump and change of direction performance to be improved in adolescent males to the same extent by either countermovement or drop jump (40 cm height) training, while sprint time was not affected [88]. Steben and Steben compared three different plyometric training types in boys and girls (age 12–14) on either vertical jump height, long jump distance or triple jumps distance and found the largest training effects for each tested parameter (drop jumps, bounding/hopping, and box drills, respectively) [73]. The principle of training specificity is further supported by the findings of Ramírez-Campillo et al. [89,90], comparing vertical plyometrics, horizontal plyometrics, and a combination of both, as well as unilateral plyometrics, bilateral plyometrics and a combination of both, respectively in pre-PHV- male soccer players. Even though vertical, horizontal and a combination did not differ statistically, vertical training was superior to a small effect compared to horizontal training for vertical jumps, and vice versa for horizontal jumps [90]. The same principle can be applied for the comparison of unilateral, bilateral and a combination of both, showing greater effect sizes for unilateral jumps after unilateral training and bilateral jumps after bilateral training, respectively [89]. Interestingly the combination (unilateral + bilateral or vertical + horizontal), elicited the greatest number of improved performance variables tested in both studies [89,90] and was therefore recommended for sports of multidirectional nature, such as soccer. Additionally the movement direction in which plyometric exercises are performed also support the principle of specificity, meaning that plyometrics in the sagital plane (e.g., squat jump, broad jump) and frontal plane (e.g., lateral hops, ice skater drill) tend to increase outcome measures to a greater degree that are performed in the same plane of motion (CMJ or lateral shuffle, respectively) [91].

Taken together, plyometric training outcomes seem to follow the principle of training specificity, and hence training has to mimic the demands of everyday or sport-specific activities.

### How training type influences physical fitness outcomes

Differences between different types of training, such as strength, power, plyometric and complex training on training-induced physical fitness performance have been examined in several studies. For instance, Lephart et al. could not observe differences in strength outcomes (e.g., leg extension/ flexion strength at 60°/s) after either eight weeks of resistance (plus balance and mobility) training or four weeks of resistance (plus balance and mobility) followed by four weeks of plyometric and resistance training in female adolescents [95]. However, they noted a change in EMG-activity of the medial hamstring for the plyometric group, which implies that plyometrics may further improve muscular activation patterns. Nielsen and co-workers compared the effects of isometric resistance vs. plyometric vs. sprint training on strength, jump and sprint performances in female youth aged 7–19 [94]. They found resistance training to elicit greater changes in strength (MVC knee extension), whereas plyometric training increased power (vertical jump height) to a larger extent. None of these training regimes were able to improve sprint performance though. In another study, Behringer et al. found that plyometric training but not resistance training improved mean service velocity of junior tennis players [96]. However, differences between training groups regarding 10 RM strength gains were not observed. Surprisingly Vom Heede et al. found a body weight/ elastic band resistance training to improve jump height more than a plyometric/ power training in male and female children [42]. Of note, the latter improved situp, pushup, pullup-hold and long jump performances to a larger extent. It has to be mentioned that this study did not report any statistics in terms of significances and therefore, results have to be viewed with caution.

Training for muscular power (Olympic weight lifting) has also been compared with traditional resistance training (“power lifting”) in male adolescents [97]. Even though no differences were observed in vertical jump improvement between groups, effect sizes indicated that power training might provide some advantage for increasing jump performance. Moreover, Chaouachi et al. compared traditional resistance training with training for muscular power (Olympic weight lifting) and plyometric training in pre-PHV male wrestlers and judo athletes [98]. They found Olympic weight lifting to produce larger jump (e.g., CMJ) and sprint (e.g., 20-m spint) improvements than plyometric training and traditional resistance training (isokinetic power at 300°/s). Further, plyometrics outperformed traditional resistance training in terms of balance and sprint performances as well as peak isokinetic torque at 60°/s and 300°/s. Of note, traditional resistance training produced larger performance improvements compared with plyometrics in peak isokinetic torque at 60°/s. In another study, De Hoyo et al. reported differences between different types of training in adolescent soccer players [99]. Performing squats with fast speed at loads of 40–60% of the 1RM resulted in larger improvements in 10-20m sprint times compared to plyometric and resisted sprint training. Additionally, the resisted sprint training resulted in smaller improvements of 30–50 m sprint times compared to the resistance training. No differences for countermovement jump height were noted between all training methods.

Escamilla and co-workers examined the impact of either strength (throwers ten), power (pneumatic resistance) or plyometric training (medicine balls) and found all training types to be equally effective in increasing the throwing velocity in adolescent baseball players [100].

Adding a power/plyometric training to resistance training in male adolescent baseball players has been shown to further increase strength gains (3RM rotational strength), specific to the additional stimulus (rotational medicine ball exercises) [101]. Also Faigenbaum et al. found the combination of strength and plyometric training (complex training) to be more effective than resistance training in combination with static stretching, for the long jump, ball toss and shuttle run in around PHV males [93]. It should be noted, that the observed difference can likely be attributed to the additional plyometric stimuli, and not due to a possible detrimental effect of the stretching. Studies with adults [141–144] and adolescents [145] show no detrimental and even some positive effects regarding strength gains or jump performance after chronic stretching. In contrast Piazza et al. found the increase of countermovement jump height to be similar, after either strength or specific weight training (complex training), consisting of either dumbbell exercises or a mix of strength and plyometric exercise with weighted belts (6% body mass), respectively in girls [92]. In the same study the resistance training improved flight times during a hopping test whereas the complex training led to shorter contact times. Lloyd et al. examined strength, plyometric and complex training in pre- and post-PHV boys and found the most outcomes being positively affected by plyometric and complex training (pre-PHV) or complex training (post-PHV) [30]. In contrast, Radnor et al., who had a similar study design, found complex training to be the most effective modality independent of maturation [37].

The comparison of strength, plyometric and power training, suggests that power training might lead to the best outcomes if a single training type is practiced, since no inferior effects compared to strength and plyometric training have been observed. A recent review and meta-analysis that compared the effects of traditional resistance training with “power” training (plyometrics) in youth population [9], observed an effect of specificity. That is, greater improvements in jump height due to plyometrics, as well as greater improvements in strength and sprint measures due to resistance training. The results from comparative studies are too inconsistent to fully support these observation, with some results being fully [42] or partly (10RM gains: [96]; maximal velocity in pre-PHV children: [37]) dissonant for the comparison of strength and plyometric training outcomes. Additionally power training compared to plyometric training shows larger effects for both sprint [98,99] and jump [97,98] performance in the youth and should therefore be further differentiated from plyometric training (“power training” in the meta-analysis by Behm et al. [9]). Finally, the above-mentioned studies seem to indicate that a combination of strength and plyometric training is likely to elicit the most improvements, especially if multiple outcome parameters are tested, and is therefore recommended from a practical standpoint.

#### How alternative resistance training methods influence proxies of muscle strength, power and speed

A growing body of literature indicates that alternative training stimuli, such as whole body vibration [146], neuromuscular stimulation [147], and blood flow restriction [148] are effective to improve muscle performance in adults. However, only few studies are available that examined the effects of these training types in youth.

For instance Mahieu et al. compared whole body vibration training with an equivalent resistance training in young (vibration: 12.9±1.5 years, resistance: 11.8±1.8 years) male and female alpine skiers [102]. The researchers found vibration to elicit larger performance enhancements in “explosive strength”/muscular endurance (the “high box test”, jumping for 90s) and plantar flexor strength (peak torque at 30°/s) compared to resistance training. However, the enrolled participants were not controlled for their maturity status, and consequently they significantly differed in age with the vibration training group being significantly older. Therefore, these results are most likely biased due to maturational effects. Moreover, the resistance training program consisted of the same exercise as the vibration training program. The only difference was that during resistance training, exercises were performed on the floor without any additional weight. Even though perceived exertion did not differ between groups, it would be interesting to see traditional resistance training with additional weights being compared. Another study combined whole body vibration and functional eccentric overload training and compared it to a strength/power/plyometric training in adolescent soccer players [103]. They found measures of speed and power (e.g. change of direction performance and mean rebounding jump height) to be affected more with the vibration/ eccentric training. Unfortunately, the study design does not allow identification of the impact of vibration, as too many variables differed between the studied groups. Nonetheless, in children and adolescents with disabilities, whole body vibration is stated to be a safe training modality that likely elicits positive effects on muscle strength [149]. Therefore future research should investigate this training modality on youth athlete and non-athlete populations.

Neuromuscular electrical stimulation (NMES) has only scarcely been applied in youth. In one study, Deley et al. investigated its effect on different performance parameters in prepubertal gymnasts, and this study revealed promising results (e.g., increased strength and jump performances) [150]. However, many studies are needed to clarify if the positive effects that has been reported in adults [147,151] can be transferred to the immature organism. Similarly, blood flow restriction (BFR) has been shown to elicit several positive muscular adaptations in adult athletes and non-athletes, combined with a low risk for negative side effects [148,152]. Although this training method has been known for over 30 years now, research for the youth population in that field is still lacking. Based on the fact that the immature nervous system seems to be unable to recruit all fast twitch fibers, both training types (NMES and BFR) may be of particular interest in that life segment, as they alter the recruitment pattern.

A recent meta-analysis [153] on the use variable resistance training, a modality where bands or chains are attached to alter the kinematics of a lift, found greater improvements of mean strength for trained and untrained adults compared to traditional resistance training. To our knowledge, only one study, published by Riviere et al., has compared this method in adolescent cohorts [104]. The authors report greater improvements for strength (relative bench press strength), power (e.g., bench press absolute mean power at 65% 1RM), and velocity (e.g., bench press mean velocity at 65% 1RM) for the variable resistance group.

Taken together all of the above mentioned modern training types have been sparsely investigated in youth population and might be promising alternative resistance training methods for improving physical fitness in children and adolescents. Future research needs to determine their safety and effects on youth populations.

## Conclusions

In line with previous reviews [1,12] and meta-analyses [6,9,23], the present systematic literature review revealed that resistance and plyometric training are effective in increasing a wide range of physical fitness outcomes in youth.

The analysis of comparative studies further indicated that maturity affects strength and plyometric training adaptations differently, with the former showing smaller and the latter showing larger effects during the pre-PHV period. Sex does not seem to impact resistance training outcomes differently. The impact of sex on plyometric training related outcomes is less clear. Pre-PHV boys and girls seem to respond equally, whereas around-PHV boys appear to show larger jump performance improvements than girls. However, research is scarce and this needs further clarification.

With regards to training descriptors in resistance training, no final conclusions can be drawn from comparative studies in terms of the optimal dose-response relations in training intensity and volume. While low training intensity / volume were recommended for beginners and untrained youth to begin with [1], more recent studies with young athletes recommend higher intensities (>80% 1RM) and volumes (5 sets) [23]. It seems though that comparative studies in resistance trained athletes suggest that “the more the better” might not be the appropriate approach to induce large gains in muscle strength. In contrast, the limited number of articles on plyometric training suggest, that low-to-moderate training volumes are effective in improving components of physical fitness. However, more research is needed in young athletes. Notably, comparative studies on the effects of rest intervals in youth resistance training are not available. The few plyometric training studies suggest that neither inter-day nor inter-set rests affect training outcomes differently. Furthermore, two sessions per week of resistance training seem to be superior compared to one session per week. Again, more research is needed to evaluate if higher training frequencies produce better results. To date, there is no information available in the literature on the effects of training frequency in youth plyometric training. The literature on the impact of periodization during resistance training in youth is also limited. The identified studies show no clear difference between no, linear or undulating models of periodization.

Comparative studies have shown that different types of strength and plyometric training can be effective. They seem to follow the principle of training specificity and should therefore be designed with respect to the children’s/ young athletes’ needs. When comparing these training types against one another, no clear picture evolves. Nevertheless, a combination of training types will likely result in the largest training-induced improvements in physical fitness. Recently, Behm et al. postulated that resistance training should be incorporated at an early age and prior to power/plyometric training in order to establish an adequate foundation of strength for power training activities [9].

Finally, we identified several research gaps in the literature which were further described in detail in each section. More specifically, training descriptors (e.g., rest intervals for resistance training), as well as modern alternative training types (e.g., NMES) need to be addressed in future comparative studies. Finally, to allow a context-related and meaningful interpretation of the reported data, comparative studies in pediatric research should (i) always report data on maturity status, (ii) modulate a single training protocol variable only in the experimental compared to the (active) control group only (e.g., exercise stimulus), (iii) provide a detailed description of the applied exercise stimulus (e.g., volume, intensity, workload), (iv) always report participants’ training history and status.

## Supporting information

S1 Checklist Prisma checklist 2009. (DOC)
