# Correction: A systematic review on the effects of resistance and plyometric training on physical fitness in youth- What do comparative studies tell us?

**DOI:** 10.1371/journal.pone.0207641

**Published:** 2018-11-14

**Authors:** Matti Peitz, Michael Behringer, Urs Granacher

There are errors in the Funding section. The correct funding information is as follows: This study is part of the research project “Resistance Training in Youth Athletes” that was funded by the German Federal Institute of Sport Science (ZMVI1-08190114-18). In addition, we acknowledge the support of the Deutsche Forschungsgemeinschaft (DFG) and Open Access Publishing Fund of University of Potsdam, Germany. The funders had no role in study design, data collection and analysis, decision to publish, or preparation of the manuscript.
